# Contribution of infection to mortality in people with type 2 diabetes: a population-based cohort study using electronic records

**DOI:** 10.1016/j.lanepe.2024.101147

**Published:** 2024-11-27

**Authors:** Iain M. Carey, Julia A. Critchley, Umar A.R. Chaudhry, Stephen DeWilde, Elizabeth S. Limb, Liza Bowen, Selma Audi, Derek G. Cook, Peter H. Whincup, Naveed Sattar, Arshia Panahloo, Tess Harris

**Affiliations:** a Population Health Research Institute, St George's, University of London, London, SW17 0RE, United Kingdom; b School of Cardiovascular and Metabolic Health, BHF Glasgow Cardiovascular Research Centre, University of Glasgow, 126 University Place, Glasgow, G12 8TA, United Kingdom; c St George's University Hospitals NHS Foundation Trust, Blackshaw Road, Tooting, London, SW17 0QT, United Kingdom

**Keywords:** Infections, Mortality, Type 2 diabetes, Sepsis, Cause of death

## Abstract

**Background:**

While people with type 2 diabetes (T2D) are more susceptible to infections, studies potentially underestimate the true burden of infection-related mortality since they rely on clinical coding systems primarily structured by body system, and by only focusing on underlying cause. This study examined cause-specific mortality in people with T2D compared to the general population during 2015–2019, focusing on infections.

**Methods:**

509,403 people aged 41–90 years with T2D alive on 1/1/2015 in Clinical Practice Research Datalink were matched to 976,431 without diabetes on age, sex, and ethnicity. Recorded underlying cause of death was identified through national linked mortality data; infection-related deaths were counted across all ICD-10 (10th revision of the International Classification of Diseases) chapters, not just infection chapters A00-B99. All-cause and cause-specific hazard ratios (HR) for mortality during 2015–2019 compared people with T2D to people without diabetes and were estimated using Cox models adjusting for region. Additional analyses for sepsis related mortality considered the impact of including any mention of sepsis on the death certificate.

**Findings:**

85,367/509,403 (16.8%) people with T2D died during 2015–2019 compared to 106,824/976,431 (10.9%) of people without diabetes of the same sex, age and ethnicity. All infections (11,128/85,367 = 13.0%) represented the third highest underlying cause of death among people with T2D after cardiovascular disease and cancer; a much higher contribution than counting only from specific infection chapters (1046/85,367 = 1.2%). The HR for people with T2D vs non-diabetes for all infection mortality (1.82, 95% CI 1.78–1.86) was higher than that estimated for all-cause (HR = 1.65, 95% CI 1.64–1.66). The estimated mortality rate associated with sepsis among people with T2D was highly dependent on whether any mention was included (2.2 per 1000 person-years) or only underlying cause (0.2 per 1000 person-years); but the HR for people with T2D vs non-diabetes was similar (any mention HR = 2.26, 95% CI 2.19–2.34 vs underlying cause only HR = 2.52, 95% CI 2.27–2.80).

**Interpretation:**

People with T2D die from infections at a higher rate than similar people without diabetes, and the overall burden is greater than previously reported. Routine statistics concentrating on underlying cause of death may somewhat under-estimate the importance of infections as causes of death among people with T2D. These findings emphasise the potential importance of awareness, earlier diagnosis and treatment of infections to prevent premature deaths.

**Funding:**

10.13039/501100000272National Institute for Health and Care Research.

**Research in context**

**Evidence before this study**

We searched PubMed for population-based analyses of infection-related causes of death in individuals with Type 2 diabetes mellitus (T2D) from 1 Jan 2010 to 21 May 2024 using the terms “infection-related mortality” and “diabetes mellitus”. We included population-based studies from high income countries reporting infection-related mortality prior to 2020, as patterns were disrupted by the COVID-19 pandemic. Infection-related mortality is an established, but rarely reported, cause of death among people with T2D. An earlier meta-epidemiological review of 97 prospective cohort studies (820,900 people) found the risk of infection-related mortality among people with diabetes more than doubled (HR [Hazard Ratio] = 2.39, 95% CI 1.95–2.93). Although the specific infections associated with this increased mortality was not explored, subsequent studies from Australia and the U.S. have reported specific findings for pneumonia, septicaemia and osteomyelitis. Few studies have been sufficiently large to stratify by age and sex, or report on diabetes mortality for people from different ethnicities, and none have reported how infection specified mortality might vary across different ethnic groups.

**Added value of this study**

This study highlights the importance of infections when assessing the increased absolute mortality risk among people with T2D. Deaths from pneumonia and urinary tract infections are common but are usually reported in other ICD categories (e.g., respiratory diseases). These deaths are infection-related and, if counted as such, then deaths from infections as an underlying cause recorded on the death certificate represent the third largest group after cardiovascular disease and cancer respectively, accounting for about 13% of all deaths among people with T2D. The HR for infection-related mortality over a 5-year period was double that of people without diabetes, and this was comparable to the HR of cardiovascular mortality at all ages. Under age 60 the HR for infection-related mortality was nearly four times higher for people with T2D compared to people without diabetes. HRs were similar across all ethnic groups but absolute differences greater for White ethnicities. As some infections, particularly sepsis, are rarely coded as underlying cause, the true impact of infections of diabetes-related mortality may still be substantially underestimated when only counting the underlying cause.

**Implications of all the available evidence**

Infection-related deaths are a common cause of death among people with T2D, and the excess relative risk is similar to that associated with vascular deaths in people with diabetes. Overall, the risk of dying from an infection with diabetes is roughly doubled, but the HR was much higher among younger people with T2D. These infections are typically thought to be preventable or treatable and this emphasises the importance of awareness of infection risk, earlier diagnosis, and appropriate prompt treatment, where possible, to prevent premature death. However, most major guidelines make little mention of infections, or how to manage or prevent them, among people with T2D.

## Introduction

People with diabetes are more susceptible to infections,^1^ and at a greater risk of all-cause mortality, than the general population.^2^^,^^3^ While there has been some reporting of an increase in infection-related mortality among people with diabetes,^3^, ^4^, ^5^, ^6^ recent large population studies of cause-specific mortality have tended to focus on other more commonly recorded underlying causes of death such as cardiovascular and cancer.^7^, ^8^, ^9^ One of the difficulties in reporting infections as a separate cause of death may be due to the hierarchal structure of ICD-10 codes which are organised into body system-related chapters,^10^ and thus infection-related causes are found across multiple chapters. Common infectious causes such as pneumonia tend to be summarised as respiratory or combined with influenza.^11^ By contrast, the specific chapters of ICD-10 dedicated to infectious disease (A00-B99) focus on communicable or transmissible disease (e.g., tuberculosis) and are comparatively rare in terms of all infections. Additionally, since recorded underlying cause of death has been acknowledged to emphasise chronic disease,^12^ solely relying on it rather than also considering the proximal cause to count deaths associated with infection can produce a considerable underestimate.^13^ This is particularly the case for sepsis, which is known to be an increasing reason for hospitalisation among people with diabetes,^14^ and while often life-threatening, is rarely listed as a cause of death.

While the COVID-19 (Coronavirus disease 2019) pandemic highlighted both the greater infection mortality risk among people living with diabetes,^15^ and related ethnic disparities,^16^ it is important to also consider the risks of infection-related mortality prior to 2020 among people living with type 2 diabetes (T2D). There is limited evidence from England that non-white ethnicities with T2D had lower mortality rates compared to the White population,^17^ similar to overall population trends,^18^ but specific patterns with infection-related mortality among people with T2D by ethnicity are less clear.^17^ Given the substantial number of younger people estimated to be diagnosed with T2D in England,^19^ summarising higher risks of infection-related mortality is potentially informative where many of these deaths could be classified as being avoidable.^20^

Therefore, this cohort study using a large primary care database in England linked to national death certification data sought to do the following: (i) Compare the rates of all-cause and cause-specific mortality between people living with T2D compared to people without diabetes in a 5-year period prior to the COVID-19 pandemic, (ii) Provide estimates of infection-related mortality by including all infection codes, (iii) Explore how these associations with mortality for T2D varied by age, sex, and ethnicity, and by type of infection, (iv) Estimate the extent to which the incidence of sepsis, around the time of death may be hidden by focusing on the recorded underlying cause only.

## Methods

### Data sources

CPRD (Clinical Practice Research Datalink) is a primary care database in the UK jointly sponsored by the Medicines and Healthcare products Regulatory Agency and the National Institute for Health and Care Research.^21^ It provides a pseudonymised longitudinal medical record for all registered patients (>99% of the UK population are registered with a General Practitioner), with diagnoses and other clinical information recorded using Read Codes. This study used a February 2022 extract from the CPRD Aurum database, which included approximately 16 million currently registered patients from 1447 general practices (England only). Over 90% of contributing practices in Aurum have consented to their data being linked to external sources, and researchers have no access to geographical identifiers such as residential postcode.^22^ These data sources include the ONS (Office for National Statistics) mortality data which includes cause of death and the Index of Multiple Deprivation (IMD), a composite small-area (approximately 1500 people) measure used in England for allocation of resources, which provides a good proxy for individual socio-economic deprivation.

### Study design and participants

The study was a matched cohort design comparing people with and without diabetes, described previously.^23^ Briefly, all patients aged 18–90 with a Read code for diabetes who were active in CPRD on 1st January 2015 and had been registered for at least one year were first identified, and classified into Type 1, Type 2 or unknown based on their diagnosis codes and anti-diabetes medication (Figure S1). For this analysis of mortality, the focus was people with T2D ages 41 and above as there are comparatively few deaths at younger ages. In the U.K., ethnicity is predominantly self-reported in primary care records and recorded using Read codes. As per previous work, four broad ethnicity categories were used (White, South Asian, Black, mixed/other), with any remaining patients without a recording (approximately 10%) classified as missing.^23^ A comparison group without known diabetes, were randomly matched on age, sex and ethnicity, with a maximum of two people without diabetes selected for each person with T2D (94% of match-sets having two comparators, 6% having one). For the main analysis, this resulted in 509,403 people with T2D and 976,431 matched non-diabetes people (Figure S1).

### Cause of death classifications

Cause of death during 2015–2019 was determined from the underlying cause code (ICD-10) in the linked ONS mortality data. The following cause-specific categories were chosen: cancer, cardiovascular, dementia (including Alzheimer's), diabetes, digestive, infections and respiratory (Table S1). All other causes, were grouped as “other”, including 158 (0.1%) deaths during 2015–2019 who had no cause recorded. For classifying deaths from infectious causes, a previous list developed for identifying infection-related hospital admissions was used,^23^ which counts across all ICD-10 chapters (codes provided in Supplementary Material). Thus, in this categorisation, pneumonia (J12-J18) is included here and not among respiratory deaths for example. Similarly, “chronic obstructive pulmonary disease with acute lower respiratory infection” (J44.0) or “with acute exacerbation, unspecified” (J44.1), and “urinary tract infection, site not specified” (N39.0) were all categorised as infection-related deaths rather than by their ICD-10 chapter. A sub-group analysis by infection type considered the following groups: bone and joint, gastrointestinal tract, genitourinary, lower respiratory tract (pneumonia or other), sepsis, and skin/cellulitis. All other potential groups had too few occurrences to be analysed separately and were classed as “other infections”.

Following earlier concerns around ICD-10 coding on death certification data resulting in sepsis deaths being underestimated,^13^ sepsis mortality was reported in two ways (i) underlying cause only, (ii) underlying or contributory. While sepsis was often a contributory cause for a non-infective underlying cause such as cancer, it did also appear where the underlying cause was also an infection e.g., cellulitis. Thus, in cause-specific analysis of infections it would be possible for a death to be classed as both skin/cellulitis (underlying) and sepsis (contributory only).

### Statistical analysis

Cox proportional hazards models were used to estimate hazard ratios (HR) for 5-year mortality in people with T2D vs those without diabetes during 2015–2019 (PROC PHREG, SAS version 9.4). These were stratified on each match-set which allows each match-set to have its own baseline hazard function, and implicitly adjusts for the matched factors (age, sex and ethnicity). Practice region (nine areas in England) was additionally adjusted for in the main analysis. Sensitivity analyses investigated, (i) the additional impact of adjusting for area deprivation (IMD quintiles) and smoking (using categories of never, current, ex or not recorded), (ii) Poisson regression using generalized estimating equations (GEE) to account for the clustering by matched set (PROC GENMOD, SAS version 9.4). For all-cause mortality, separate models were additionally fitted to 10-year age groups, including for men and women individually, while in sensitivity analyses, we also fitted the overall model to each individual age separately. All study participants were followed up from 1st January 2015 to 31st December 2019, or their linked date of death if it occurred before then. In further sensitivity analyses, earlier censoring of follow-up time was applied to, (i) patients who de-registered from their practice during 2015–2019, (ii) patients without diabetes on 1st January 2015 who were diagnosed with diabetes during 2015–2019.

To contrast the estimates from the Cox model, which provide a *relative comparison* (of the instantaneous mortality rate during follow-up among those still alive),^24^ the *absolute difference* in overall crude mortality rates (annually per 1000 person years of follow-up time) was also estimated by subtracting the non-diabetes rate from the T2D rate. People with non-diabetes in each match-set were weighted according to whether there were 1 or 2 in each match-set. 95% confidence intervals for the difference in rates were estimated using Stata (version 15).

The analysis was repeated within the four ethnic groups, but with wider age groups (41–60, 61–75, 76–90) to account for smaller numbers among non-white ethnicities. For analyses of mortality by underlying cause, cause-specific models were used which treat causes other than ones of interest as censored and allows the usual interpretation of the hazard ratio.^24^ These were fitted to all study participants, and by age group (41–60, 61–75, 76–90 for all causes, and 41–75, 76–90 for the analysis of infection-specific causes).

### Role of the funding source

The study funder had no role in study design, collection, analysis, and interpretation of data; writing of the report or decision to submit the paper for publication. The corresponding author had full access to all the data in the study and final responsibility for the decision to submit for publication.

## Results

A summary of the baseline characteristics of the 509,403 people with T2D, and the 976,431 without diabetes is provided in Table S2. Among T2D, 56% were men with a mean age = 67.3 years (SD = 11.9). People with T2D were more likely to have a BMI of 30 or more recorded (50% vs 22%) and be living in the most deprived quintile (23% vs 16%) than people without diabetes. About a third (34%) of people with T2D had been diagnosed in the last 5 years (Table S3).

During 2015–2019, a total of 85,367 (16.8%) people with T2D aged 41–90 died (Table 1), compared to 10.9% of the matched non-diabetes group (HR = 1.65, 95% CI 1.64–1.66). Within 10-year age groups, the HR ranged from HR = 2.95 (95% CI 2.75–3.17) in ages 41–50 to HR = 1.39 (95% CI 1.38–1.41) in ages 81–90. Fitting the model to individual ages highlighted that the HR was nearer 4 at around age 40, and about 1.20 at age 90 (Figure S2). While the HRs comparing to their respective non-diabetes group were consistently higher for women overall (HR = 1.71), and within different age groups, the corresponding absolute differences in mortality rate between people with T2D and people without diabetes were quite similar for men and women (13.1 vs 13.9 per 1000 person-years). Table 2 summarises all-cause mortality for people with T2D by recorded ethnicity. Within each age-group, the absolute differences in the mortality rate between people with T2D vs people without diabetes of the same ethnicity were consistently higher for the White group. While the largest HR overall was observed for the South Asian group (HR = 1.73, 95% CI 1.66–1.80), age-specific comparisons reveal that the HR was generally higher among White ethnicity, particularly at 41–60 years (HR = 2.57, 95% CI 2.48–2.67). Sensitivity analyses that investigated the impact of different censoring of participants during follow-up (Table S4), using Poisson regression (Table S5) or further adjusting for deprivation and smoking (Table S6) did not meaningfully change the results.

**Table 1:** All-cause mortality during 2015–2019 in people with T2D vs non-diabetes.

Sex	Age group	Type 2 diabetes (T2D)				Non-diabetes				T2D vs non-diabetes	
		N	Died	%	Mortality rate^a^	N	Died	%	Mortality rate^b^	Difference in rates^c^ (95% CI)	HR^d^ (95% CI)
All	All ages	509,403	85,367	16.8	36.6	976,431	106,824	10.9	23.2	13.4 (13.2–13.7)	1.65 (1.64–1.66)
	41–50	50,393	1230	2.4	4.9	99,746	833	0.8	1.7	3.3 (3.0–3.6)	2.95 (2.75–3.17)
	51–60	103,229	4875	4.7	9.7	203,683	3934	1.9	3.9	5.8 (5.5–6.1)	2.46 (2.38–2.54)
	61–70	141,183	13,892	9.8	20.7	269,813	13,601	5.0	10.2	10.5 (10.1–10.8)	2.03 (1.99–2.07)
	71–80	137,642	29,664	21.6	48.3	257,754	34,925	13.6	28.6	19.6 (19.0–20.3)	1.70 (1.68–1.72)
	81–90	76,956	35,706	46.4	122.5	145,435	53,531	36.8	89.1	33.4 (31.9–34.9)	1.39 (1.38–1.41)
Women	All ages	224,720	37,125	16.5	36.0	430,819	45,162	10.5	22.1	13.9 (13.5–14.3)	1.71 (1.69–1.73)
	41–50	21,167	489	2.3	4.7	41,913	295	0.7	1.4	3.3 (2.8–3.7)	3.35 (2.97–3.78)
	51–60	41,748	1788	4.3	8.7	82,638	1303	1.6	3.2	5.6 (5.1–6.0)	2.72 (2.57–2.88)
	61–70	58,135	5026	8.7	18.1	111,065	4300	3.9	7.8	10.3 (9.7–10.8)	2.33 (2.26–2.41)
	71–80	63,106	12,144	19.2	42.5	118,188	13,201	11.2	23.3	19.2 (18.4–20.1)	1.84 (1.80–1.87)
	81–90	40,564	17,678	43.6	112.4	77,015	26,063	33.8	80.3	32.0 (30.1–34.0)	1.42 (1.40–1.45)
Men	All ages	284,683	48,242	17.0	37.1	545,612	61,662	11.3	24.0	13.1 (12.7–13.4)	1.61 (1.59–1.62)
	41–50	29,226	741	2.5	5.1	58,833	538	0.9	1.9	3.3 (2.9–3.7)	2.75 (2.51–3.00)
	51–60	61,481	3087	5.0	10.3	121,045	2631	2.2	4.4	5.9 (5.5–6.3)	2.33 (2.24–2.43)
	61–70	83,048	8866	10.7	22.5	158,748	9301	5.9	11.9	10.6 (10.1–11.1)	1.89 (1.85–1.94)
	71–80	74,536	17,520	23.5	53.3	139,566	21,724	15.6	33.2	20.0 (19.1–20.9)	1.62 (1.59–1.64)
	81–90	36,392	18,028	49.6	134.3	68,420	27,468	40.2	99.3	35.0 (32.7–37.3)	1.36 (1.34–1.39)

a Crude mortality rates per 1000 person-years.

b Crude mortality rates per 1000 person-years weighted according to number of persons (1 or 2) in matched set.

c Absolute difference in rates between T2D and non-diabetes.

d Hazard ratio from Cox model stratified by age-sex-ethnicity matched set and additionally adjusted for region.

**Table 2:** All-cause mortality during 2015–2019 in people with T2D vs non-diabetes, by ethnicity

Ethnicity	Age group	Type 2 diabetes (T2D)				Non-diabetes				T2D vs non-diabetes	
		N	Died	%	Mortality rate^a^	N	Died	%	Mortality rate^b^	Difference in rates^c^ (95% CI)	HR^d^ (95% CI)
South Asian	All ages	49,579	3734	7.5	15.7	77,418	2686	3.5	9.4	6.3 (5.8–6.9)	1.73 (1.66–1.80)
	41–60	24,078	513	2.1	4.3	47,160	421	0.9	1.8	2.5 (2.1–2.9)	2.27 (2.04–2.52)
	61–75	18,473	1340	7.3	15.1	22,895	857	3.7	8.1	7.0 (6.1–7.9)	1.83 (1.71–1.96)
	76–90	7028	1881	26.8	62.5	7363	1408	19.1	41.8	20.7 (17.5–24.0)	1.51 (1.43–1.59)
Black	All ages	21,328	2045	9.6	20.2	36,604	1871	5.1	14.0	6.2 (5.2–7.2)	1.48 (1.41–1.56)
	41–60	10,013	232	2.3	4.7	19,793	291	1.5	3.0	1.7 (1.0–2.4)	1.58 (1.38–1.82)
	61–75	6417	543	8.5	17.7	11,385	549	4.8	10.5	7.2 (5.5–8.9)	1.66 (1.50–1.83)
	76–90	4898	1270	26.0	60.0	5426	1031	19.0	43.8	16.2 (12.4–20.1)	1.38 (1.29–1.47)
Mixed	All ages	28,491	2378	8.4	17.5	52,334	2390	4.6	11.2	6.2 (5.4–7.0)	1.63 (1.55–1.70)
	41–60	12,764	265	2.1	4.2	25,260	255	1.0	2.0	2.2 (1.6–2.7)	2.12 (1.84–2.44)
	61–75	10,535	752	7.1	14.8	19,926	766	3.8	8.2	6.6 (5.4–7.8)	1.81 (1.66–1.96)
	76–90	5192	1361	26.2	60.7	7148	1369	19.2	42.8	17.9 (14.2–21.6)	1.44 (1.35–1.53)
White	All ages	360,312	62,762	17.4	38.1	712,714	81,524	11.4	24.2	13.9 (13.6–14.2)	1.63 (1.61–1.64)
	41–60	94,225	4166	4.4	9.0	186,578	3234	1.7	3.5	5.5 (5.2–5.8)	2.57 (2.48–2.67)
	61–75	157,650	19,260	12.2	26.0	311,896	20,490	6.6	13.6	12.4 (12.0–12.8)	1.91 (1.88–1.94)
	76–90	108,437	39,336	36.3	88.9	214,240	57,800	27.0	62.2	26.7 (25.6–27.7)	1.45 (1.43–1.47)

a Crude mortality rates per 1000 person-years.

b Crude mortality rates per 1000 person-years weighted according to number of persons (1 or 2) in matched set.

c Absolute difference in rates between T2D and non-diabetes.

d Hazard ratio from Cox model stratified by age-sex-ethnicity matched set and additionally adjusted for region. Mean age (years) of people with T2D in subgroups: **All:** South Asian (61.6), Black (63.5), Mixed (63.1), White (68.3); **41–60:** South Asian (51.9), Black (52.0), Mixed (52.8), White (53.1); **61–75:** South Asian (67.0), Black (68.0), Mixed (67.6), White (68.3); **76–90:** South Asian (80.7), Black (80.9), Mixed (80.9), White (81.5). Note: People with no ethnicity recorded (10%) are not included in the above table.

**Fig. 1:**
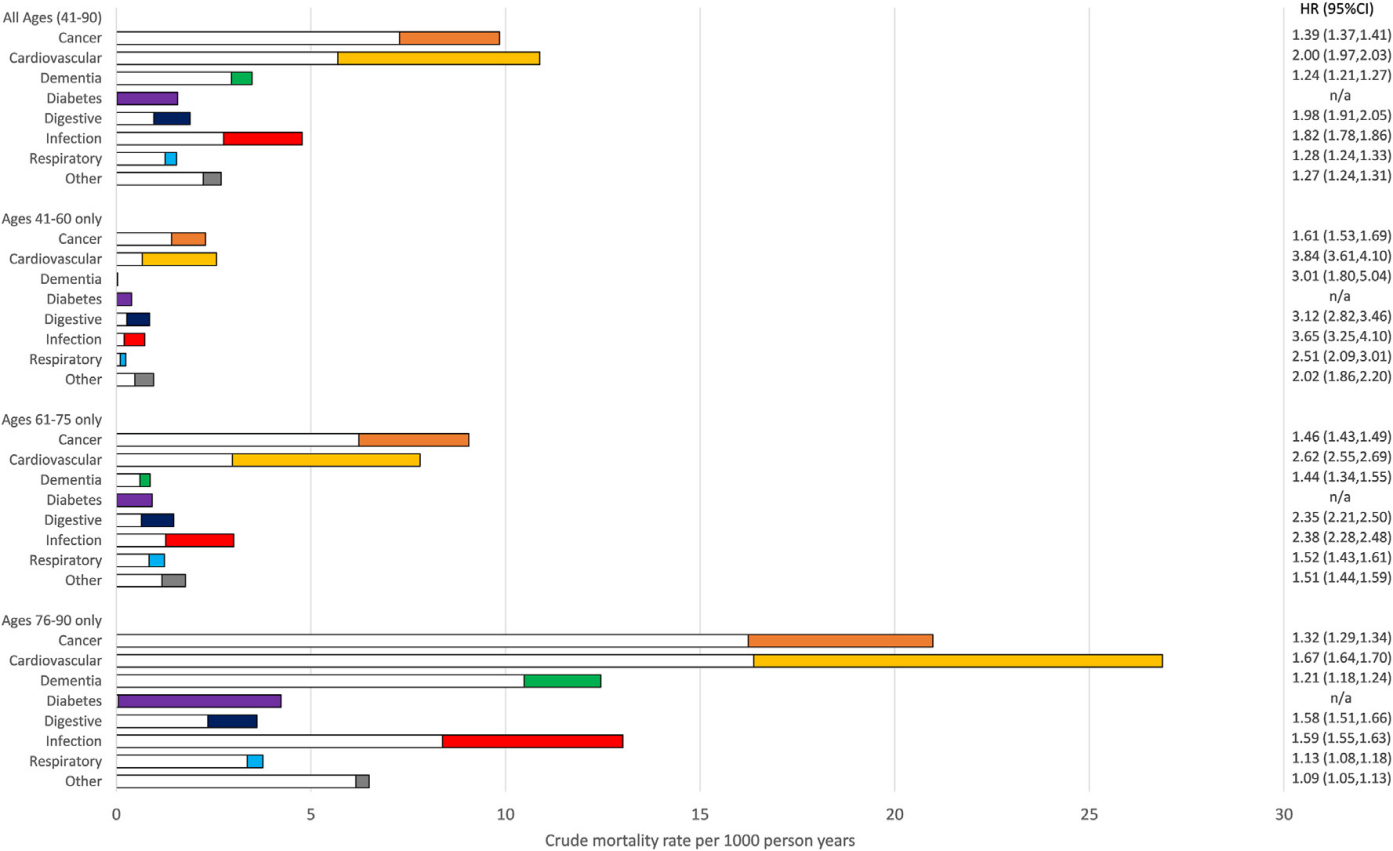
Crude mortality rates and hazard ratios by cause of death during 2015–2019 in people with T2D vs non-diabetes. Footnote: White bars = crude mortality rate in non-diabetes weighted according to number of persons (1 or 2) in matched set. Coloured bars = excess crude mortality rate in people with T2D. Combined bar (white + colour) = crude mortality rate in people with T2D. LRT = Lower Respiratory Tract.

Among all deaths in the study (Figure S3), the top 3 causes were cardiovascular (T2D = 29.7%, non-diabetes = 24.4%), cancer (T2D = 26.9%, non-diabetes = 31.6%) and all infections including pneumonia (T2D = 13.0%, non-diabetes = 12.0%). By contrast, the proportion of deaths from the infection-specific chapters (A00-B99) were much smaller (T2D = 1.2%, non-diabetes = 1.0%). Fig. 1 summarises crude mortality rates and hazard ratios by underlying cause of death in people with T2D vs non-diabetes (data provided in Table S7). The largest HR for all ages was seen for cardiovascular mortality (HR = 2.00, 95% CI 1.97–2.03), which also had the greatest difference in mortality rate (represented by the coloured bar). The highest HRs among the other causes, were digestive (1.98 95% CI 1.91–2.05) and infections (1.82, 95% CI 1.78–1.86), while cancer and infections contributed significant excess mortality (denoted by the length of the coloured bars in the figure). A sensitivity analysis only counting infection deaths from ICD-10 chapters A00-B99 (Table S8), estimated that the crude mortality rate was approximately twice as high among people with T2D vs non-diabetes (0.45 vs 0.22 per 1000 person years), with an estimated HR = 2.09 (95% CI 1.95–2.25). In the main analysis among the younger age groups (Fig. 1), the HRs were consistently high for cardiovascular and all infectious causes (HR = 3.84 and 3.65 for 41–60 years, HR = 2.62 and 2.38 for 61–75 years respectively). Repeating the analysis by ethnicity (Figure S4), showed the HR for infection mortality was consistent in all ethnic groups (HRs between 1.71 and 1.82).

The extent to which infections appear overall on death certificates (underlying cause plus any mention) is summarised in Figure S5 for people with T2D and without diabetes. Among those who died, almost 1-in-3 people with T2D (31.0%) have an infection coded anywhere on the certificate (29.8% in non-diabetes). A significant proportion listed sepsis elsewhere as a contributing cause where the underlying cause was non-infective (3.2% T2D, 2.5% non-diabetes). Sensitivity analyses that included any mention of sepsis as an infection death, marginally increased the HR from 1.82 in Fig. 1 to 1.88 (95% CI 1.84–1.91). Additionally, among people with T2D there were also 477 deaths (0.6%) where diabetes was the underlying cause, but an infection other than sepsis was listed as a contributing cause.

**Fig. 2:**
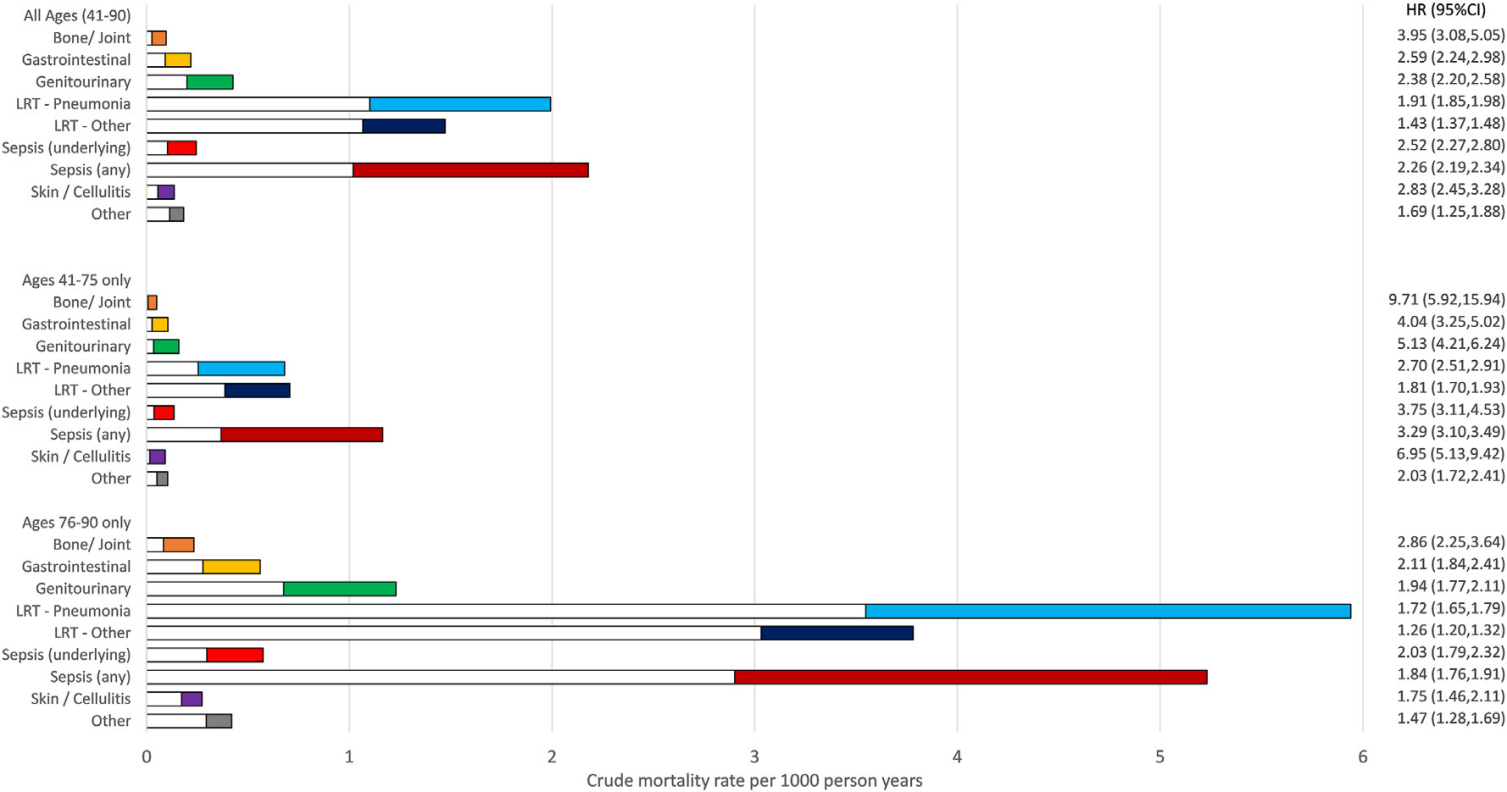
Crude mortality rates and hazard ratios for different infectious causes of death during 2015–2019 in people with T2D vs non-diabetes. Footnote: White bars = crude mortality rate in non-diabetes weighted according to number of persons (1 or 2) in matched set. Coloured bars = excess crude mortality rate in people with T2D. Combined bar (white + colour) = crude mortality rate in people with T2D. LRT = Lower Respiratory Tract.

Finally, mortality associations were investigated by type of infection recorded as an underlying cause of death during 2015–2019 (Table S9). Fig. 2 summarises the cause-specific mortality rates and HRs for different infection groupings in people with T2D vs non-diabetes (with accompanying data in Table S10). Overall, the highest HR was observed for bone and joint infections (HR = 3.95, 95% CI 3.08–5.05). The largest difference in mortality rates between T2D and non-diabetes (length of the coloured bars) was seen for lower respiratory tract (LRT), particularly pneumonia. The estimated mortality rates for sepsis were vastly different when any mention on the death certificate was considered (T2D = 2.2 per 1000 person-years, non-diabetes = 1.0 per 1000 person-years) compared to underlying cause only (T2D = 0.2 per 1000 person-years, non-diabetes = 0.1 per 1000 person-years). Put differently, among all T2D deaths with any mention of sepsis on the death certificate (n = 5076), only 11% (n = 570) had it recorded as the underlying cause. When the analysis was re-run including any mention of sepsis on the death certificate, the excess in mortality rate was comparable to the LRT/pneumonia group, while the hazard ratio remained high (HR = 2.26 vs 2.52 for underlying cause only). Among younger people with T2D, there were high HRs for bone and joint infections (HR = 9.71) and skin/cellulitis (HR = 6.95), although these were rare occurrences.

## Discussion

This study used large electronic health databases to highlight the higher risk of dying from infections for people with T2D, from a period just prior to the COVID-19 pandemic. Reports of infectious causes of death using ICD-10 are generally summarised using the “Certain infectious and parasitic diseases” chapters (A00-B99).^10^ However, as an underlying cause of death these are comparatively rare. Additionally, in the UK a recent change in coding rules for cause of death estimated a fall in 2017 of almost 20% of deaths with an underlying cause of death from these two chapters.^25^ More common infective causes of death such as a urinary tract infection, will generally be counted by researchers under their respective main ICD-10 chapter (e.g., genitourinary system). By using a more inclusive set of ICD-10 codes to collate them, infections were then estimated to be the third highest contributor to mortality (after cardiovascular disease and cancers) during 2015–2019, accounting for about 13% of deaths overall, compared to only 1.2% when standard ICD-10 categories are used. The HR of infection-related death was about double that for people without diabetes, and this was similar when infections were defined using only the A00-B99 chapters. Among younger people with T2D (41–60 years), infection-related mortality was almost 4 times higher than among the general population, comparable to excess deaths from cardiovascular causes.

Population-based studies from the UK,^7^^,^^17^ Denmark^9^^,^^26^ and the US^11^ have compared cause-specific mortality for infections among T2D vs people without diabetes. These have consistently found elevated risks but most report only on risk associated with specific infections, or only use the A00-B99 chapters to summarise infections. For example, data from NHANES in the US has found that the risk of influenza and pneumonia-specific mortality was 3.5 times higher in people with T2D.^11^ The present analyses differ from the recent UK comprehensive review of causes of death in T2D by Pearson-Stuttard et al.,^7^ which only included infections as part an “other” category or classified them elsewhere (pneumonia as respiratory for example). They reported that cancer mortality was the most common cause of death in people with T2D, reflecting changing patterns in mortality, with declining vascular deaths in the whole population, resulting in cancer deaths becoming more common as a proportion of total deaths. The present analysis highlighted the excess risk associated with specific causes of death for people with T2D, showing that the excess risk of cardiovascular death among people with T2D was still likely to be greater than for any other cause, and excess mortality risks associated with infection were potentially greater than those of cancer.

One of the main strengths of the present analysis, is the large sample size (half a million people with T2D), that enabled an ethnicity matched comparison group without diabetes, addressing limitations of earlier CPRD analyses of mortality and T2D.^17^ This approach accounted for the ethnic specific variations in the prevalence of T2D as well as differences in the age structure between the older white population and non-white ethnicities. Although about 10% of people with T2D could not be assigned an ethnicity, previous work has shown these people tended to be older and more affluent,^23^ resembling the characteristics of the White ethnicity group, suggesting that a high proportion of non-White ethnicities with T2D have been identified. After stratifying mortality by ethnicity, there was a greater estimated excess mortality risk for people with T2D of White ethnicity compared with other non-White ethnic groups, particularly in the youngest age group. This agrees with the findings from Wright et al., who estimated that incident T2D among White ethnicity was associated with more years of life lost compared to South Asians or Black ethnic group,^17^ and showed that people of White ethnicity tend to develop T2D at higher BMI levels compared to people of South Asian and Black ethnicity.^27^ It is also of note that there is now evidence that obesity is itself associated with infections^28^ and that among all people with T2D, younger people tend to have greater obesity levels.^27^

A potential limitation of the present analysis was only including people aged 41–90 at the start of the follow-up period in 2015 in the cohort to enable the ethnicity matching. The exclusion of 40 years and under is negligible in terms of 5-year mortality. Although the prevalence of T2D declines above age 90,^29^ by not including the oldest ages the work underestimates the burden of dementia as a cause of death. Nevertheless, this analysis includes the overwhelming majority of preventable deaths occurring. Another limitation of the analysis is that by utilising the linked death certification data for an analysis that ignored loss to follow-up, patients who de-registered from their general practice and died during 2015–2019 would only be captured in the linkage if they died in England. However, even if emigration is related to ethnicity in our cohort population, matching on ethnicity should largely negate this. Thus, while the analysis may have underestimated some absolute mortality risks, the relative risks (or hazard ratios) would not be affected.

While no other studies to our knowledge have similarly aggregated infection-related mortality, some have highlighted that infection-related mortality may be underestimated. An earlier US analysis comparing Medicare claims and in-hospital mortality among the whole population found that death certificates frequently overlooked infections as both proximal and contributory causes of death, and tend to focus on long-term conditions and chronic disease.^12^ When this current analysis used conventional ICD-10-chapter classifications, among people with T2D who died over a 5-year period, only about 1% died due to an infectious cause, but using the more inclusive reclassification increased this to around 13%. Our previous analysis showed that hospitalisations where the admission episode is due to an infection is very common among people with T2D; over 20% had an infection-related hospitalisation over a 5-year time-period.^23^ Given this relatively high hospitalisation rate, it could be argued that conventional ICD-10 classification overlooks the importance of infection-related causes of death in people with T2D. Although the COVID-19 pandemic highlighted higher mortality rates among non-white ethnicities in the UK,^16^ there was little evidence to suggest that these same groups were at higher risk prior to 2020 for infection related mortality. This might reflect the lower risk of death among non-white ethnicities prior to 2020,^18^ as well as the different causal pathways through which ethnicity can influence contagious and other disease. More recent research from Scotland has highlighted that both COVID-19 and non-COVID-19 pneumonias increase the risk of premature cardiovascular death in people with T2D,^30^ supporting plentiful earlier research that suggested any serious infection can temporarily increase these risks as systemic inflammation stresses the cardiovascular system.^31^

Additionally, it is of interest that treatment with semaglutide (a GLP-1 agonist) in people with obesity and cardiovascular disease, but without diabetes, in the SELECT trial was associated with significantly lower rates of death from cardiovascular causes compared with placebo.^32^ UK guidelines recommend the introduction of these drugs for people with T2D with obesity and cardiovascular disease.^33^ Prescribing of SGLT-2 inhibitors is also likely to rise^34^; these anti-diabetes drugs reduce cardiovascular risk but may predispose patients to urine and genital infections.^35^ Whilst prescribing of these drugs was low in our dataset (5% of T2D receiving GLP-1 or SGLT-2), increases in their use have the potential to further reduce the morbidity and mortality of cardiovascular disease in T2D, while making infections more prominent as a cause of death. However, at present guidelines for people with T2D provide little emphasis on infection risks or management.^33^

Apart from sepsis, this current analysis only assessed the underlying cause of death and did not consider the contributory role of infections. Had it looked further at contributory or proximal causes of death, about 31% of death certificates in people with T2D mention infection. This issue is most pertinent when it comes to sepsis related deaths, as these are rarely coded as the underlying cause.^13^ Moreover, recent coding changes have occurred in the UK leading to more deaths being counted with an immediate rather than underlying cause of sepsis.^25^ We are not aware of any evidence that this change would impact people with diabetes differently from people without diabetes, and sensitivity analyses that included any mention of sepsis produced similar findings compared to counting underlying cause only. Sepsis is a complex and serious public health issue with a rising prevalence.^36^ Additionally, people living with diabetes are more likely to be hospitalised with sepsis,^14^ and have been estimated to have a four times higher case-fatality for sepsis.^37^ In this study over a 5-year period approximately 1% of all people with T2D who died had sepsis on their death certificate, but only one-in-ten of these deaths listed sepsis as the underlying cause. Regardless of whether sepsis was listed as the underlying cause of death, people with T2D had both larger absolute and relative risks of sepsis mortality compared to non-diabetes, especially among those under age 75 years. A recent systematic case series of preventable coroners’ reports for sepsis deaths in England and Wales estimated an average of 17.5 years of life was being lost, and more could be done to improve sepsis care and patient safety.^38^

From a public health perspective, excess death in terms of premature mortality is associated with higher economic costs and social burden. The World Health Organisation defines early mortality, often quantified by a metric such as years of life lost from mortality, as death that occurs at an age younger than the average expected lifespan for a specific population.^39^ While there is evidence that the average individual years of life lost from T2D has been reducing over time, the burden across the population is still rising.^40^ Therefore, excess deaths from infections in people with T2D, especially at a younger age (<60 years), will impact productivity (labour force participation, economic output, societal contributions) as well as result in financial and emotional strain on families and communities. Prevention strategies that can address infection risk among people with T2D have the potentially to significantly reduce premature mortality.

In conclusion, our study suggests whether underlying or contributory, infection-related deaths are common major causes or contributors to diabetes-related mortality. Despite this, the risk of infections is barely mentioned in T2D guidelines. Increased awareness by both patients and clinicians might enhance health seeking behaviour, result in prompter diagnosis and preventative treatment, and reduce the risk of serious infections that can be fatal.

## Contributors

Iain Carey: Conceptualization, Data curation, Formal analysis, Investigation, Methodology, Writing—original draft, Writing–review & editing. Julia Critchley: Conceptualization, Funding acquisition, Methodology, Project administration, Writing—original draft, Writing–review & editing. Umar Chaudhry: Methodology, Writing–review & editing. Stephen DeWilde: Methodology, Writing–review & editing. Elizabeth Limb: Methodology, Writing–review & editing. Liza Bowen: Methodology, Writing–review & editing. Selma Audi: Methodology, Writing–review & editing. Derek Cook: Conceptualization, Data curation, Methodology, Writing—original draft, Writing–review & editing. Peter Whincup: Methodology, Writing–review & editing. Naveed Sattar: Conceptualization, Writing–review & editing. Arshia Panahloo: Conceptualization, Writing–review & editing. Tess Harris: Conceptualization, Funding acquisition, Data curation, Methodology, Project administration, Writing—original draft, Writing–review & editing.

All authors approved the final version for publication, and IC had final responsibility for the decision to submit.

## Data sharing statement

The study is based on electronic health data from the Clinical Practice Research Datalink (CPRD) obtained under license from the UK Medicines and Healthcare Products Regulatory Agency (MHRA). CPRD data governance and the license to use CPRD data does not allow distribution of patient data directly to other parties. Researchers must apply directly to CPRD for data access (https://www.cprd.com).

## Declaration of interests

NS has consulted for and/or received speaker honoraria from Abbott Laboratories, AbbVie, Amgen, AstraZeneca, Boehringer Ingelheim, Eli Lilly, Hanmi Pharmaceuticals, Janssen, Menarini-Ricerche, Novartis, Novo Nordisk, Pfizer, Roche Diagnostics, and Sanofi; and received grant support paid to his University from AstraZeneca, Boehringer Ingelheim, Novartis, and Roche Diagnostics outside the submitted work.

## Appendix A Supplementary data

Supplementary Material Revised
