# Supplementary material for: Contribution of infection to mortality in people with type 2 diabetes: a population-based cohort study using electronic records
Source: Lancet Reg Health Eur. 2024 Nov 27;48:101147. doi: 10.1016/j.lanepe.2024.101147 (PMC11634975; doi:10.1016/j.lanepe.2024.101147)

Supplementary Material

Contents

[Table S1 – ICD-10 codes for main underlying causes of death 2](#_Toc182981802)

[Table S2 – Baseline characteristics of patients with and without type 2 diabetes 3](#_Toc182981803)

[Table S3 – Medication and diagnosis history of patients with type 2 diabetes 4](#_Toc182981804)

[Table S4 – Sensitivity analyses restricting follow-up time for all-cause mortality during 2015-9 in T2D vs non-diabetes, all people 5](#_Toc182981805)

[Table S5 – Poisson regression (RR) vs. Cox regression (HR) estimates for all-cause mortality during 2015-9 in T2D vs non-diabetes, all people and by sex 6](#_Toc182981806)

[Table S6 – Further adjusted hazard ratios for all-cause and selected cause-specific mortality during 2015-9 in T2D vs non-diabetes 7](#_Toc182981807)

[Table S7 – Crude annual mortality rates during 2015-9 by different underlying causes in T2D and non-diabetes 8](#_Toc182981808)

[Table S8 – Number of deaths and crude mortality rates by ICD-10 chapter 9](#_Toc182981809)

[Table S9 – Deaths during 2015-9 with infection as underlying cause by ICD-10 code 10](#_Toc182981810)

[Table S10 – Crude annual mortality rates during 2015-9 by different underlying infectious causes in T2D and non-diabetes 14](#_Toc182981811)

[Figure S1 – Summary of selected study participants with type 2 diabetes, and matched group without any diabetes 15](#_Toc182981812)

[Figure S2 – Age-specific hazard ratios and risk ratios for all-cause mortality during 2015-9 in T2D vs non-diabetes, all patients 16](#_Toc182981813)

[Figure S3 – Cause of death during 2015-9 in T2D and non-diabetes, by different categorisations 17](#_Toc182981814)

[Figure S4 – Crude mortality rates and hazard ratios by cause of death during 2015-9 in T2D vs non-diabetes by ethnicity 18](#_Toc182981815)

[Figure S5 – Percentage of deaths during 2015-9 with infection recorded as underlying cause, any mention of sepsis or infection as contributory cause only in T2D vs non-diabetes, ages 41-90. 19](#_Toc182981816)

## Table S1 – ICD-10 codes for main underlying causes of death

| Group | ICD-10 Codes |
| --- | --- |
| Cancer | C00–D49 |
| Cardiovascular (excluding infections) | I05–I99  except I30.1, I32.0. I32.1, I33.0, I33.9, I38, I39.8, I40, I41.0, I41.1, I41.2, I43.0, I98.0, I98.1 |
| Dementia  (including Alzheimer’s) | F01, F03,  G30, G31 |
| Diabetes | E10–E14 |
| Digestive | K20–K94  except K12.2, K23.0, K23.1, K61, K63.0, K67.3, K75.0, K77.0, K80.0, K80.1, K80.4, K81 |
| Infections | A00–B20, B25–B88,  G00–G08,  H00.0, H10.0, H10.2, H10.3, H10.5, H13.1, H13.2, H16.1, H16.2, H16.3, H16.8, H16.9, H19.1, H19.2, H19.3, H44.0, H60.0, H60.1, H60.2, H60.3, H60.4, H60.8, H60.9, H62.0, H62.1, H62.2, H62.3, H62.4, H65.0, H65.1, H66.0, H66.4, H66.9, H67, H70, H73.0, H73.1, H75.0,  I00–I02, I30.1, I32.0. I32.1, I33.0, I33.9, I38, I39.8, I40, I41.0, I41.1, I41.2, I43.0, I98.0, I98.1,  J00–J06, J10.0, J11.0, J12–J22, J34.0, J36, J39.0, J39.1, J44.0, J44.1, J65, J85.0, J85.1, J85.2, J85.3, J86.0, J86.9,  K12.2, K23.0, K23.1, K61, K63.0, K67.3, K75.0, K77.0, K80.0, K80.1, K80.4, K81,  L00–L08, L30.3, L66.3, L66.4, L73.9,  M00–M01, M46.2, M46.3, M46.4, M46.5, M86, M90.1, M90.2, N08.0, N10, N11.0, N11.1, N12, N13.6, N15.1, N15.9, N16.0, N29.0, N29.1, N30.0, N30.8, N30.9, N33.0, N34, N37.0, N39.0, N41.0, N41.2, N41.3, N43.1, N45, N48.1, N70–N72, N73.0, N73.1, N73.2, N73.8, N73.9, N74.0, N74.1, N75.1, N77.1,  O85,  T79.3, T80.2, T81.4, T82.6, T82.7, T83.5, T83.6, T84.5, T84.6, T84.7, T85.7, T87.4, T88.0,  U04.9, U80, U81, U88, U89, Z21 |
| Respiratory  (excluding infections) | J09–J11, J30–J99  except J10.0, J11.0, J34.0, J36, J39.0, J39.1, J44.0, J44.1, J65, J85.0, J85.1, J85.2, J85.3, J86.0, J86.9 |

## Table S2 – Baseline characteristics of patients with and without type 2 diabetes

|  | | People with T2D (n=509,403) | | Non-diabetes (n=976,431) | |
| --- | --- | --- | --- | --- | --- |
|  | | N | % | N | % |
|  |  |  |  |  |  |
| Sex | Females | 224,720 | 44.1% | 430,819 | 44.1% |
|  | Males | 284,683 | 55.9% | 545,612 | 55.9% |
|  |  |  |  |  |  |
| Age (in 2015) | 41 to 50 | 50,393 | 9.9% | 99,746 | 10.2% |
|  | 51 to 60 | 103,229 | 20.3% | 203,683 | 20.9% |
|  | 61 to 70 | 141,183 | 27.7% | 269,813 | 27.6% |
|  | 71 to 80 | 137,642 | 27.0% | 257,754 | 26.4% |
|  | 81 to 90 | 76,956 | 15.1% | 145,435 | 14.9% |
|  |  |  |  |  |  |
| Ethnicity | South Asian | 49,579 | 9.7% | 77,418 | 7.9% |
|  | Black | 21,328 | 4.2% | 36,604 | 3.8% |
|  | Mixed | 28,491 | 5.6% | 52,334 | 5.4% |
|  | White | 360,312 | 70.7% | 712,714 | 73.0% |
|  | Missing | 49,693 | 9.8% | 97,361 | 10.0% |
|  |  |  |  |  |  |
| Region | East Midlands | 11,344 | 2.2% | 22,913 | 2.4% |
|  | East of England | 20,038 | 3.9% | 49,381 | 5.1% |
|  | London | 60,185 | 11.8% | 118,018 | 12.1% |
|  | North East | 17,855 | 3.5% | 35,394 | 3.6% |
|  | North West | 97,701 | 19.2% | 171,730 | 17.6% |
|  | South Central | 92,300 | 18.1% | 207,131 | 21.2% |
|  | South West | 99,353 | 19.5% | 174,741 | 17.9% |
|  | West Midlands | 93,247 | 18.3% | 164,214 | 16.8% |
|  | Yorkshire & Humber | 17,380 | 3.4% | 32,909 | 3.4% |
|  |  |  |  |  |  |
| Index of Multiple Deprivation* | 1 (least deprived) | 85,862 | 16.9% | 230,823 | 23.6% |
|  | 2 | 95,369 | 18.7% | 215,803 | 22.1% |
|  | 3 | 98,636 | 19.4% | 191,986 | 19.7% |
|  | 4 | 112,305 | 22.1% | 180,425 | 18.5% |
|  | 5 (most deprived) | 116,989 | 22.9% | 156,607 | 16.0% |
|  | Missing | 362 | 0.1% | 787 | 0.1% |
|  |  |  |  |  |  |
| Body Mass Index | <25 | 78,917 | 15.5% | 332,763 | 34.1% |
|  | 25 to <30 | 171,803 | 33.7% | 369,292 | 37.8% |
|  | 30 to <40 | 212,384 | 41.7% | 192,981 | 19.8% |
|  | ≥40 | 43,531 | 8.6% | 16,444 | 1.7% |
|  | Not recorded | 2,768 | 0.5% | 64,951 | 6.7% |
|  |  |  |  |  |  |
| Smoking | Never | 188,733 | 37.1% | 435,116 | 44.6% |
|  | Ex | 248,899 | 48.9% | 396,301 | 40.6% |
|  | Current | 71,273 | 14.0% | 132,931 | 13.6% |
|  | Not recorded | 498 | 0.1% | 12,093 | 1.2% |
|  |  |  |  |  |  |
| Number of  co-morbidities‡ | 0 | 119,290 | 23.4% | 511,117 | 52.4% |
|  | 1-2 | 310,841 | 61.0% | 399,872 | 41.0% |
|  | >2 | 79,272 | 15.6% | 65,442 | 6.7% |

* - A composite small-area (approximately 1500 people) measure used in England for allocation of resources - <https://www.gov.uk/government/collections/english-indices-of-deprivation>
‡ - Count of the following: Atrial fibrillation, cancer, chronic obstructive pulmonary disease, coronary heart disease, chronic kidney disease, dementia, epilepsy, heart failure, hypertension, peripheral vascular disease, serious mental Illness (e.g. psychosis, schizophrenia & bipolar affective disorder), stroke/TIA.

## Table S3 – Medication and diagnosis history of patients with type 2 diabetes

|  |  | N | % |
| --- | --- | --- | --- |
|  |  |  |  |
| All |  | 509,403 | 100% |
|  |  |  |  |
| Time Since Diagnosis | 0 to 5 years | 173,585 | 34.1% |
|  | 5 to 15 yrs. | 250,431 | 49.2% |
|  | >15 years | 85,387 | 16.8% |
|  |  |  |  |
| Summary of all anti-diabetic medications prescribed in 2014 | None | 116,280 | 22.8% |
|  | Biguanides only | 23,192 | 4.6% |
|  | Biguanides & other^1^ | 156,819 | 30.8% |
|  | Other^2^ | 141,571 | 27.8% |
|  | Insulin^3^ | 71,541 | 14.0% |
|  |  |  |  |
| Specific anti-diabetic medications prescribed in 2014 | Biguanides | 349,342 | 68.6% |
|  | Sulphonylureas | 148,602 | 29.2% |
|  | Gliptins | 71,921 | 14.1% |
|  | Insulin | 71,541 | 14.0% |
|  | Thiazolidinediones | 22,475 | 4.4% |
|  | GLP-1s | 17,354 | 3.4% |
|  | Gliflozins or SGLT2s | 7,886 | 1.6% |
|  | Meglitinides | 1,641 | 0.3% |
|  | Alpha glucosidase inhibitors | 912 | 0.2% |

1 - Excluding insulin, 2 - Excluding biguanides & insulin, 3 - Only or in combination

## Table S4 – Sensitivity analyses restricting follow-up time for all-cause mortality during 2015-9 in T2D vs non-diabetes, all people

| Restriction | Type 2 diabetes (T2D)  N=509,403 | | Non-diabetes N=976,431 | | T2D vs non-diabetes | | |
| --- | --- | --- | --- | --- | --- | --- | --- |
|  | Died | Rate per 1,000 PY^1^ | Died | Rate per 1,000 PY^1^ | Rate Difference^2^ | HR† | 95%CI |
|  |  |  |  |  |  |  |  |
| No restriction on follow-up time  (Main analysis, Table 1) | 85,367 | 36.6 | 106,824 | 23.2 | 13.4 | 1.65 | 1.64-1.66 |
| Censored people if they de-register from CPRD practice | 76,599 | 35.5 | 94,353 | 22.2 | 13.3 | 1.66 | 1.64-1.67 |
| Censor patients without diabetes earlier if they are diagnosed with diabetes^3^ | 76,599 | 35.5 | 92,049 | 22.0 | 13.5 | 1.67 | 1.65-1.68 |
| 1-year mortality only, all data censored at 31/12/2015 | 16,466 | 32.9 | 19,581 | 20.3 | 12.6 | 1.63 | 1.60-1.66 |

1 - Crude mortality rate per 1,000 person years of follow-up. Note that the rate for non-diabetes is weighted depending on whether 1 or 2 non-diabetes persons are in the age-sex-ethnicity matched set.
2 - Difference in crude mortality rates (T2D minus non-diabetes) per 1,000 person years of follow-up.
3 – A total of 2,204 people without diabetes (0.2%) were diagnosed with diabetes during follow-up
†HR - Hazard ratio from Cox model, stratified by age-sex-ethnicity matched set. Additionally adjusted for region.

## Table S5 – Poisson regression (RR) vs. Cox regression (HR) estimates for all-cause mortality during 2015-9 in T2D vs non-diabetes, all people and by sex

| Sex | Age group | Poisson Regression | | Cox Model (Table 1) | |
| --- | --- | --- | --- | --- | --- |
|  |  | RR* | 95%CI | HR† | 95%CI |
|  |  |  |  |  |  |
| All | All ages | 1.62 | 1.61-1.64 | 1.65 | 1.64-1.66 |
|  |  |  |  |  |  |
|  | 41-50 | 2.93 | 2.68-3.20 | 2.95 | 2.75-3.17 |
|  | 51-60 | 2.47 | 2.36-2.57 | 2.46 | 2.38-2.54 |
|  | 61-70 | 2.02 | 1.98-2.07 | 2.03 | 1.99-2.07 |
|  | 71-80 | 1.70 | 1.68-1.73 | 1.70 | 1.68-1.72 |
|  | 81-90 | 1.39 | 1.37-1.41 | 1.39 | 1.38-1.41 |
|  |  |  |  |  |  |
| Women | All ages | 1.67 | 1.65-1.70 | 1.71 | 1.69-1.73 |
|  |  |  |  |  |  |
|  | 41-50 | 3.30 | 2.86-3.82 | 3.35 | 2.97-3.78 |
|  | 51-60 | 2.75 | 2.56-2.95 | 2.72 | 2.57-2.88 |
|  | 61-70 | 2.32 | 2.22-2.41 | 2.33 | 2.26-2.41 |
|  | 71-80 | 1.83 | 1.79-1.88 | 1.84 | 1.80-1.87 |
|  | 81-90 | 1.42 | 1.39-1.45 | 1.42 | 1.40-1.45 |
|  |  |  |  |  |  |
| Men | All ages | 1.59 | 1.57-1.60 | 1.61 | 1.59-1.62 |
|  |  |  |  |  |  |
|  | 41-50 | 2.73 | 2.44-3.05 | 2.75 | 2.51-3.00 |
|  | 51-60 | 2.33 | 2.21-2.45 | 2.33 | 2.24-2.43 |
|  | 61-70 | 1.89 | 1.83-1.95 | 1.89 | 1.85-1.94 |
|  | 71-80 | 1.62 | 1.59-1.65 | 1.62 | 1.59-1.64 |
|  | 81-90 | 1.37 | 1.34-1.40 | 1.36 | 1.34-1.39 |
|  |  |  |  |  |  |

* - Relative risk (and 95% confidence intervals) from GEE Poisson model, for mortality between T2D and non-diabetes accounting for clustering by match set, adjusted for age, sex, ethnicity and region.
† - Hazard ratio from Cox model, stratified by match set, fitted to age groups separately. Additionally adjusted for region

## Table S6 – Further adjusted hazard ratios for all-cause and selected cause-specific mortality during 2015-9 in T2D vs non-diabetes

|  | + Region (Main analysis) | | + Region & Deprivation | | + Region & Smoking | | + Region, Deprivation & Smoking | |
| --- | --- | --- | --- | --- | --- | --- | --- | --- |
|  | HR† | 95%CI | HR† | 95%CI | HR† | 95%CI | HR† | 95%CI |
|  |  |  |  |  |  |  |  |  |
| All-cause |  |  |  |  |  |  |  |  |
| All patients | 1.65 | 1.64-1.66 | 1.61 | 1.59-1.62 | 1.61 | 1.60-1.63 | 1.58 | 1.57-1.59 |
|  |  |  |  |  |  |  |  |  |
| Women | 1.71 | 1.69-1.73 | 1.65 | 1.63-1.67 | 1.67 | 1.65-1.69 | 1.63 | 1.61-1.65 |
| Men | 1.61 | 1.59-1.62 | 1.57 | 1.56-1.59 | 1.57 | 1.55-1.59 | 1.54 | 1.53-1.56 |
|  |  |  |  |  |  |  |  |  |
| Ages 41-50 | 2.95 | 2.75-3.17 | 2.67 | 2.48-2.87 | 2.89 | 2.68-3.11 | 2.69 | 2.49-2.90 |
| Ages 51-60 | 2.46 | 2.38-2.54 | 2.30 | 2.22-2.38 | 2.43 | 2.34-2.52 | 2.31 | 2.22-2.39 |
| Ages 61-70 | 2.03 | 1.99-2.07 | 1.92 | 1.89-1.96 | 1.99 | 1.95-2.03 | 1.91 | 1.87-1.95 |
| Ages 71-80 | 1.70 | 1.68-1.72 | 1.65 | 1.63-1.67 | 1.66 | 1.64-1.68 | 1.62 | 1.60-1.64 |
| Ages 81-90 | 1.39 | 1.38-1.41 | 1.38 | 1.36-1.39 | 1.37 | 1.36-1.39 | 1.36 | 1.34-1.38 |
|  |  |  |  |  |  |  |  |  |
| South Asian | 1.73 | 1.66-1.80 | 1.70 | 1.63-1.77 | 1.70 | 1.64-1.77 | 1.68 | 1.61-1.75 |
| Black | 1.48 | 1.41-1.56 | 1.47 | 1.40-1.55 | 1.48 | 1.41-1.56 | 1.48 | 1.40-1.56 |
| Mixed | 1.63 | 1.55-1.70 | 1.59 | 1.51-1.66 | 1.64 | 1.56-1.72 | 1.60 | 1.53-1.68 |
| White | 1.63 | 1.61-1.64 | 1.58 | 1.57-1.60 | 1.60 | 1.59-1.62 | 1.57 | 1.55-1.58 |
|  |  |  |  |  |  |  |  |  |
| Cause-specific |  |  |  |  |  |  |  |  |
| Cardiovascular | 2.00 | 1.97-2.03 | 1.95 | 1.92-1.98 | 1.97 | 1.94-2.00 | 1.92 | 1.90-1.95 |
| Infection | 1.82 | 1.78-1.86 | 1.76 | 1.72-1.80 | 1.78 | 1.74-1.83 | 1.73 | 1.70-1.77 |
|  |  |  |  |  |  |  |  |  |

† – Hazard ratio for all-cause or cause-specific mortality from Cox model stratified by match set. Deprivation was adjusted by Index of Multiple Deprivation (IMD) quintiles. Smoking was adjusted using the categories of Never, Current, Ex and Not Recorded.

## Table S7 – Crude annual mortality rates during 2015-9 by different underlying causes in T2D and non-diabetes

| Underlying | All ages (41-90) | | | Ages 41-60 | | | Ages 61-75 | | | Ages 76-90 | | |
| --- | --- | --- | --- | --- | --- | --- | --- | --- | --- | --- | --- | --- |
| Cause | T2D | Non-D | Difference | T2D | Non-D | Difference | T2D | Non-D | Difference | T2D | Non-D | Difference |
|  |  |  |  |  |  |  |  |  |  |  |  |  |
| Cancer | 9.8 | 7.3 | *+2.6* | 2.3 | 1.4 | *+0.9* | 9.1 | 6.2 | *+2.8* | 21.0 | 16.2 | *+4.7* |
| Cardiovascular | 10.9 | 5.7 | *+5.2* | 2.6 | 0.7 | *+1.9* | 7.8 | 3.0 | *+4.8* | 26.9 | 16.4 | *+10.5* |
| Dementia | 3.5 | 3.0 | *+0.5* | 0.0 | 0.0 | *+0.0* | 0.9 | 0.6 | *+0.3* | 12.5 | 10.5 | *+2.0* |
| Diabetes | 1.6 | 0.0 | *+1.6* | 0.4 | 0.0 | *+0.4* | 0.9 | 0.0 | *+0.9* | 4.2 | 0.1 | *+4.2* |
| Digestive | 1.9 | 1.0 | *+0.9* | 0.9 | 0.3 | *+0.6* | 1.5 | 0.6 | *+0.8* | 3.6 | 2.4 | *+1.3* |
| Infection | 4.8 | 2.8 | *+2.0* | 0.7 | 0.2 | *+0.5* | 3.0 | 1.3 | *+1.7* | 13.0 | 8.4 | *+4.6* |
| Respiratory | 1.6 | 1.3 | *+0.3* | 0.2 | 0.1 | *+0.1* | 1.2 | 0.8 | *+0.4* | 3.8 | 3.4 | *+0.4* |
| Other | 2.7 | 2.2 | *+0.5* | 1.0 | 0.5 | *+0.5* | 1.8 | 1.2 | *+0.6* | 6.5 | 6.2 | *+0.3* |

Note: Mortality rates are per 1000-person years for people with Type 2 diabetes (T2D) and age-sex-ethnicity matched non-diabetes (Non-D) group, and correspond to bars plotted in Figure 1 in main paper. Difference is T2D rate minus Non-D rate.

## Table S8 – Number of deaths and crude mortality rates by ICD-10 chapter

| ICD-10 Chapter | ICD-10 Codes | Type 2 Diabetes  N=509,403 | | Non-diabetes  N=976,431 | |
| --- | --- | --- | --- | --- | --- |
|  |  | n | R/1000† | n | R/1000† |
| Certain infectious and parasitic diseases | A00-B99 | 1,046 | 0.45 | 1,025 | 0.22 |
| Neoplasms | C00-D49 | 22,956 | 9.85 | 33,732 | 7.28 |
| Diseases of the blood and blood-forming organs and certain disorders involving the immune mechanism | D50-D89 | 175 | 0.08 | 202 | 0.05 |
| Endocrine, nutritional and metabolic diseases | E00-E89 | 4,060 | 1.74 | 432 | 0.10 |
| Mental, Behavioural and Neurodevelopmental disorders | F01-F99 | 6,147 | 2.64 | 9,311 | 2.03 |
| Diseases of the nervous system | G00-G99 | 3,608 | 1.55 | 7,726 | 1.67 |
| Diseases of the eye and adnexa | H00-H59 | * | <0.001 | * | <0.001 |
| Diseases of the ear and mastoid process | H60-H95 | 8 | 0.003 | 6 | 0.001 |
| Diseases of the circulatory system | I00-I99 | 25,607 | 10.98 | 26,360 | 5.76 |
| Diseases of the respiratory system | J00-J99 | 11,700 | 5.02 | 15,818 | 3.42 |
| Diseases of the digestive system | K00-K95 | 4,464 | 1.91 | 4,636 | 1.00 |
| Diseases of the skin and subcutaneous tissue | L00-L99 | 406 | 0.17 | 394 | 0.09 |
| Diseases of the musculoskeletal system and connective tissue | M00-M99 | 578 | 0.25 | 781 | 0.17 |
| Diseases of the genitourinary system | N00-N99 | 1,638 | 0.70 | 1,703 | 0.37 |
| Pregnancy, childbirth and the puerperium | O00-O9A | 0 | _ | 0 | _ |
| Certain conditions originating in the perinatal period | P00-P96 | 0 | _ | 0 | _ |
| Congenital malformations, deformations and chromosomal abnormalities | Q00-Q99 | 84 | 0.04 | 173 | 0.04 |
| Symptoms, signs and abnormal clinical and laboratory findings, not elsewhere classified | R00-R99 | 709 | 0.30 | 1,509 | 0.32 |
| Injury, poisoning and certain other consequences of external causes | S00-T88 | 0 | _ | 0 | _ |
| External causes of morbidity | V00-Y99 | 2,062 | 0.88 | 2,846 | 0.61 |
| Factors influencing health status and contact with health services | Z00-Z99 | 0 | _ | 0 | _ |
| Codes for special purposes | U00-U85 | 47 | 0.02 | 79 | 0.02 |
| Not recorded | Blank | 70 | 0.03 | 88 | 0.02 |

* - Non-zero frequency 5 or less. † - R/1000 is the crude mortality rate per 1,000 person-years, with the non-diabetes rate weighted by match-set.

ICD-10 Chapters taken from: <https://icd.who.int/browse10/2019/en>

## Table S9 – Deaths during 2015-9 with infection as underlying cause by ICD-10 code

| ICD-10 | Description | Type* | T2 Diabetes | | Non-diabetes | |
| --- | --- | --- | --- | --- | --- | --- |
|  |  |  | n | %‡ | n | %‡ |
| A02.0 | Salmonella enteritis | GIT | * | <0.04% | 0 | 0.00% |
| A02.1 | Salmonella sepsis | Sepsis | _ | _ | * | <0.04% |
| A04.5 | Campylobacter enteritis | GIT | * | <0.04% | * | <0.04% |
| A04.7 | Enterocolitis due to Clostridium difficile | GIT | 86 | 0.75% | 95 | 0.73% |
| A07.2 | Cryptosporidiosis | GIT | 0 | 0.00% | * | <0.04% |
| A08.1 | Acute gastroenteropathy due to Norwalk agent | GIT | 5 | 0.04% | * | <0.04% |
| A08.4 | Viral intestinal infection, unspecified | GIT | 11 | 0.10% | * | <0.04% |
| A09.0 | Other and unspecified gastroenteritis and colitis of infectious origin | GIT | 5 | 0.04% | 11 | 0.08% |
| A09.9 | Gastroenteritis and colitis of unspecified origin | GIT | 126 | 1.10% | 124 | 0.96% |
| A16.2 | Tuberculosis of lung, without mention of bacteriological or histological | TB | 15 | 0.13% | 18 | 0.14% |
| A16.4 | Tuberculosis of larynx, trachea and bronchus, without mention of bacteriological or histological confirmation | TB | * | <0.04% | 0 | 0.00% |
| A16.5 | Tuberculous pleurisy, without mention of bacteriological or histological confirmation | TB | * | <0.04% | 0 | 0.00% |
| A16.9 | Respiratory tuberculosis unspecified, without mention of bacteriological or histological confirmation | TB | 10 | 0.09% | 13 | 0.10% |
| A17.0 | Tuberculous meningitis | TB | * | <0.04% | * | <0.04% |
| A18.0 | Tuberculosis of bones and joints | TB | * | <0.04% | 0 | 0.00% |
| A18.2 | Tuberculous peripheral lymphadenopathy | TB | 0 | 0.00% | * | <0.04% |
| A18.3 | Tuberculosis of intestines, peritoneum and mesenteric glands | TB | * | <0.04% | 0 | 0.00% |
| A18.8 | Tuberculosis of other specified organs | TB | 0 | 0.00% | * | <0.04% |
| A19.9 | Miliary tuberculosis, unspecified | TB | 6 | 0.05% | 5 | 0.04% |
| A28.0 | Pasteurellosis | Other | * | <0.04% | 0 | 0.00% |
| A31.0 | Pulmonary mycobacterial infection | LRTI | 0 | 0.00% | * | <0.04% |
| A31.9 | Mycobacterial infection, unspecified | Other | 0 | 0.00% | 5 | 0.04% |
| A32.1 | Listerial meningitis and meningoencephalitis | CNS | * | <0.04% | 0 | 0.00% |
| A32.7 | Listerial sepsis | Sepsis | 0 | 0.00% | * | <0.04% |
| A32.9 | Listeriosis, unspecified | Other | * | <0.04% | 0 | 0.00% |
| A35 | Other tetanus | CNS | 0 | 0.00% | * | <0.04% |
| A37.0 | Whooping cough due to Bordetella pertussis | URTI | 0 | 0.00% | * | <0.04% |
| A39.0 | Meningococcal meningitis | CNS | 0 | 0.00% | * | <0.04% |
| A39.1 | Waterhouse-Friderichsen syndrome | Sepsis | 0 | 0.00% | * | <0.04% |
| A39.4 | Meningococcemia, unspecified | Sepsis | * | <0.04% | 6 | 0.05% |
| A39.8 | Other meningococcal infections | Other | 0 | 0.00% | * | <0.04% |
| A40.0 | Sepsis due to streptococcus, group A | Sepsis | * | <0.04% | 7 | 0.05% |
| A40.1 | Sepsis due to streptococcus, group B | Sepsis | 0 | 0.00% | * | <0.04% |
| A40.2 | Septicaemia due to streptococcus, group D | Sepsis | * | <0.04% | * | <0.04% |
| A40.3 | Sepsis due to Streptococcus pneumoniae | Sepsis | 0 | 0.00% | 6 | 0.05% |
| A40.8 | Other streptococcal sepsis | Sepsis | * | <0.04% | * | <0.04% |
| A40.9 | Streptococcal sepsis, unspecified | Sepsis | 13 | 0.11% | 12 | 0.09% |
| A41.0 | Sepsis due to Staphylococcus aureus | Sepsis | 16 | 0.14% | 6 | 0.05% |
| A41.2 | Sepsis due to unspecified staphylococcus | Sepsis | 16 | 0.14% | 8 | 0.06% |
| A41.3 | Sepsis due to Hemophilus influenzae | Sepsis | 0 | 0.00% | * | <0.04% |
| A41.4 | Sepsis due to anaerobes | Sepsis | 0 | 0.00% | * | <0.04% |
| A41.5 | Septicaemia due to other Gram-negative organisms | Sepsis | 36 | 0.31% | 35 | 0.27% |
| A41.8 | Other specified septicaemia | Sepsis | 6 | 0.05% | * | <0.04% |
| A41.9 | Sepsis, unspecified | Sepsis | 489 | 4.28% | 400 | 3.09% |
| A42.0 | Pulmonary actinomycosis | LRTI | * | <0.04% | 0 | 0.00% |
| A48.1 | Legionnaires' disease | LRTI | * | <0.04% | 5 | 0.04% |
| A49.0 | Staphylococcal infection, unspecified | Skin | 22 | 0.19% | 16 | 0.12% |
| A49.1 | Streptococcal infection, unspecified | Other | 9 | 0.08% | 12 | 0.09% |
| A49.3 | Mycoplasma infection, unspecified | Other | 0 | 0.00% | * | <0.04% |
| A49.8 | Other bacterial infections of unspecified site | Other | 16 | 0.14% | 18 | 0.14% |
| A49.9 | Bacterial infection, unspecified | Other | 0 | 0.00% | * | <0.04% |
| A52.3 | Neurosyphilis, unspecified | Other | 0 | 0.00% | * | <0.04% |
| A86 | Unspecified viral encephalitis | CNS | 10 | 0.09% | 15 | 0.12% |
| B00.4 | Herpesviral encephalitis | CNS | 5 | 0.04% | 7 | 0.05% |
| B00.9 | Herpesviral infection, unspecified | Skin | 0 | 0.00% | * | <0.04% |
| B01.1 | Varicella encephalitis (G05.1*) | CNS | * | <0.04% | * | <0.04% |
| B01.2 | Varicella pneumonia | LRTI | 0 | 0.00% | * | <0.04% |
| B02.0 | Zoster encephalitis | CNS | * | <0.04% | 6 | 0.05% |
| B02.3 | Zoster ocular disease | Eye | * | <0.04% | * | <0.04% |
| B02.8 | Zoster with other complications | CNS | * | <0.04% | 0 | 0.00% |
| B02.9 | Zoster without complications | Skin | 7 | 0.06% | 7 | 0.05% |
| B16.2 | Acute hepatitis B without delta-agent with hepatic coma | GIT | * | <0.04% | * | <0.04% |
| B16.9 | Acute hepatitis B without delta-agent and without hepatic coma | GIT | 6 | 0.05% | * | <0.04% |
| B17.2 | Acute hepatitis E | GIT | * | <0.04% | 0 | 0.00% |
| B18.1 | Chronic viral hepatitis B without delta-agent | GIT | * | <0.04% | 6 | 0.05% |
| B18.2 | Chronic viral hepatitis C | GIT | 32 | 0.28% | 20 | 0.15% |
| B19.9 | Unspecified viral hepatitis without hepatic coma | GIT | * | <0.04% | * | <0.04% |
| B20.0 | HIV disease resulting in mycobacterial infection | Other | * | <0.04% | * | <0.04% |
| B20.1 | HIV disease resulting in other bacterial infections | Other | * | <0.04% | * | <0.04% |
| B20.4 | HIV disease resulting in candidiasis | Mycoses | * | <0.04% | 0 | 0.00% |
| B20.6 | HIV disease resulting in Pneumocystis carinii pneumonia | Mycoses | 0 | 0.00% | * | <0.04% |
| B20.7 | HIV disease resulting in multiple infections | Other | * | <0.04% | * | <0.04% |
| B20.8 | HIV disease resulting in other infectious and parasitic diseases | Other | * | <0.04% | 5 | 0.04% |
| B25.0 | Cytomegaloviral pneumonitis | LRTI | * | <0.04% | 0 | 0.00% |
| B25.8 | Other cytomegaloviral diseases | Other | * | <0.04% | 0 | 0.00% |
| B25.9 | Cytomegaloviral disease, unspecified | Other | * | <0.04% | 0 | 0.00% |
| B27.0 | Gammaherpesviral mononucleosis | URTI | * | <0.04% | 0 | 0.00% |
| B33.2 | Viral carditis | Heart | 0 | 0.00% | * | <0.04% |
| B33.3 | Retrovirus infections, not elsewhere classified | Other | * | <0.04% | * | <0.04% |
| B34.0 | Adenovirus infection, unspecified | URTI | 0 | 0.00% | * | <0.04% |
| B34.8 | Other viral infections of unspecified site | Other | 5 | 0.04% | 6 | 0.05% |
| B34.9 | Viral infection, unspecified | Other | * | <0.04% | * | <0.04% |
| B37.1 | Pulmonary candidiasis | Mycoses | * | <0.04% | 0 | 0.00% |
| B37.4 | Candidiasis of other urogenital sites | Mycoses | * | <0.04% | 0 | 0.00% |
| B37.7 | Candidal sepsis | Sepsis | * | <0.04% | * | <0.04% |
| B37.8 | Candidiasis of other sites | Mycoses | * | <0.04% | * | <0.04% |
| B37.9 | Candidiasis, unspecified | Mycoses | * | <0.04% | 0 | 0.00% |
| B44.0 | Invasive pulmonary aspergillosis | Mycoses | * | <0.04% | * | <0.04% |
| B44.1 | Other pulmonary aspergillosis | Mycoses | 9 | 0.08% | 23 | 0.18% |
| B44.9 | Aspergillosis, unspecified | Mycoses | 0 | 0.00% | * | <0.04% |
| B45.0 | Pulmonary cryptococcosis | Mycoses | 0 | 0.00% | * | <0.04% |
| B46.0 | Pulmonary mucormycosis | Mycoses | * | <0.04% | * | <0.04% |
| B49 | Unspecified mycosis | Mycoses | * | <0.04% | * | <0.04% |
| B59 | Pneumocystosis | LRTI | 0 | 0.00% | 12 | 0.09% |
| G00.0 | Hemophilus meningitis | CNS | * | <0.04% | * | <0.04% |
| G00.1 | Pneumococcal meningitis | CNS | 8 | 0.07% | 8 | 0.06% |
| G00.8 | Other bacterial meningitis | CNS | 0 | 0.00% | * | <0.04% |
| G00.9 | Bacterial meningitis, unspecified | CNS | * | <0.04% | * | <0.04% |
| G03.9 | Meningitis, unspecified | CNS | 12 | 0.10% | 9 | 0.07% |
| G06.0 | Intracranial abscess and granuloma | CNS | * | <0.04% | 6 | 0.05% |
| G06.1 | Intraspinal abscess and granuloma | CNS | 0 | 0.00% | * | <0.04% |
| G06.2 | Extradural and subdural abscess, unspecified | CNS | 11 | 0.10% | * | <0.04% |
| G08 | Intracranial and intraspinal phlebitis and thrombophlebitis | CNS | * | <0.04% | 6 | 0.05% |
| H16.3 | Interstitial and deep keratitis | Eye | 0 | 0.00% | * | <0.04% |
| H44.0 | Purulent endophthalmitis | Eye | * | <0.04% | 0 | 0.00% |
| H60.0 | Abscess of external ear | URTI | * | <0.04% | 0 | 0.00% |
| H60.2 | Malignant otitis externa | URTI | 7 | 0.06% | 0 | 0.00% |
| H66.9 | Otitis media, unspecified | URTI | * | <0.04% | * | <0.04% |
| H70.0 | Acute mastoiditis | URTI | * | <0.04% | 0 | 0.00% |
| H70.9 | Mastoiditis, unspecified | URTI | 0 | 0.00% | * | <0.04% |
| I01.9 | Acute rheumatic heart disease, unspecified | Heart | * | <0.04% | 0 | 0.00% |
| I30.1 | Infective pericarditis | Heart | * | <0.04% | 0 | 0.00% |
| I33.0 | Acute and subacute infective endocarditis | Heart | 75 | 0.66% | 85 | 0.66% |
| I38 | Endocarditis, valve unspecified | Heart | 177 | 1.55% | 210 | 1.62% |
| I40.0 | Infective myocarditis | Heart | * | <0.04% | 5 | 0.04% |
| J02.9 | Acute pharyngitis, unspecified | URTI | * | <0.04% | 0 | 0.00% |
| J03.9 | Acute tonsillitis, unspecified | URTI | * | <0.04% | 0 | 0.00% |
| J04.1 | Acute tracheitis | URTI | 0 | 0.00% | * | <0.04% |
| J05.1 | Acute epiglottitis | URTI | * | <0.04% | * | <0.04% |
| J06.9 | Acute upper respiratory infection, unspecified | URTI | * | <0.04% | * | <0.04% |
| J10.0 | Influenza with pneumonia, influenza virus identified | LRTI | 48 | 0.42% | 51 | 0.39% |
| J11.0 | Influenza with pneumonia, virus not identified | LRTI | 49 | 0.43% | 51 | 0.39% |
| J12.0 | Adenoviral pneumonia | LRTI | 0 | 0.00% | * | <0.04% |
| J12.1 | Respiratory syncytial virus pneumonia | LRTI | * | <0.04% | 0 | 0.00% |
| J12.2 | Parainfluenza virus pneumonia | LRTI | * | <0.04% | * | <0.04% |
| J12.9 | Viral pneumonia, unspecified | LRTI | 10 | 0.09% | * | <0.04% |
| J13 | Pneumonia due to Streptococcus pneumoniae | LRTI | 20 | 0.17% | 30 | 0.23% |
| J14 | Pneumonia due to Hemophilus influenzae | LRTI | * | <0.04% | * | <0.04% |
| J15.0 | Pneumonia due to Klebsiella pneumoniae | LRTI | * | <0.04% | * | <0.04% |
| J15.1 | Pneumonia due to Pseudomonas | LRTI | * | <0.04% | 0 | 0.00% |
| J15.2 | Pneumonia due to staphylococcus | LRTI | 6 | 0.05% | * | <0.04% |
| J15.4 | Pneumonia due to other streptococci | LRTI | * | <0.04% | 6 | 0.05% |
| J15.5 | Pneumonia due to Escherichia coli | LRTI | * | <0.04% | 0 | 0.00% |
| J15.7 | Pneumonia due to Mycoplasma pneumoniae | LRTI | * | <0.04% | * | <0.04% |
| J15.9 | Unspecified bacterial pneumonia | LRTI | 0 | 0.00% | * | <0.04% |
| J18.0 | Bronchopneumonia, unspecified organism | LRTI | 1531 | 13.39% | 1727 | 13.33% |
| J18.1 | Lobar pneumonia, unspecified organism | LRTI | 211 | 1.85% | 253 | 1.95% |
| J18.2 | Hypostatic pneumonia, unspecified organism | LRTI | 16 | 0.14% | 13 | 0.10% |
| J18.9 | Pneumonia, unspecified organism | LRTI | 2901 | 25.38% | 2981 | 23.01% |
| J20.8 | Acute bronchitis due to other specified organisms | LRTI | * | <0.04% | 0 | 0.00% |
| J20.9 | Acute bronchitis, unspecified | LRTI | 19 | 0.17% | 10 | 0.08% |
| J21.9 | Acute bronchiolitis, unspecified | LRTI | * | <0.04% | * | <0.04% |
| J22 | Unspecified acute lower respiratory infection | LRTI | 594 | 5.20% | 651 | 5.02% |
| J39.0 | Retropharyngeal and parapharyngeal abscess | URTI | * | <0.04% | 0 | 0.00% |
| J39.1 | Other abscess of pharynx | URTI | * | <0.04% | 0 | 0.00% |
| J44.0 | Chronic obstructive pulmonary disease with acute lower respiratory infection | LRTI | 2247 | 19.66% | 3297 | 25.44% |
| J44.1 | Chronic obstructive pulmonary disease with (acute) exacerbation | LRTI | 582 | 5.09% | 1036 | 8.00% |
| J65 | Pneumoconiosis associated with tuberculosis | TB | * | <0.04% | 0 | 0.00% |
| J85.0 | Gangrene and necrosis of lung | LRTI | 0 | 0.00% | * | <0.04% |
| J85.1 | Abscess of lung with pneumonia | LRTI | * | <0.04% | 11 | 0.08% |
| J85.2 | Abscess of lung without pneumonia | LRTI | 9 | 0.08% | 6 | 0.05% |
| J86.0 | Pyothorax with fistula | LRTI | * | <0.04% | * | <0.04% |
| J86.9 | Pyothorax without fistula | LRTI | 31 | 0.27% | 40 | 0.31% |
| K12.2 | Cellulitis and abscess of mouth | Skin | 0 | 0.00% | * | <0.04% |
| K61.0 | Anal abscess | GIT | * | <0.04% | * | <0.04% |
| K61.1 | Rectal abscess | GIT | * | <0.04% | 0 | 0.00% |
| K61.3 | Ischiorectal abscess | GIT | * | <0.04% | * | <0.04% |
| K63.0 | Abscess of intestine | GIT | * | <0.04% | * | <0.04% |
| K75.0 | Abscess of liver | GIT | 29 | 0.25% | 18 | 0.14% |
| K80.0 | Calculus of gallbladder with acute cholecystitis | GIT | 20 | 0.17% | 6 | 0.05% |
| K80.1 | Calculus of gallbladder with other cholecystitis | GIT | 15 | 0.13% | 9 | 0.07% |
| K80.4 | Calculus of bile duct with cholecystitis | GIT | * | <0.04% | 0 | 0.00% |
| K81.0 | Acute cholecystitis | GIT | 63 | 0.55% | 59 | 0.46% |
| K81.1 | Chronic cholecystitis | GIT | * | <0.04% | * | <0.04% |
| K81.9 | Cholecystitis, unspecified | GIT | 99 | 0.87% | 57 | 0.44% |
| L02.1 | Cutaneous abscess, furuncle and carbuncle of neck | Skin | * | <0.04% | * | <0.04% |
| L02.2 | Cutaneous abscess, furuncle and carbuncle of trunk | Skin | * | <0.04% | * | <0.04% |
| L02.3 | Cutaneous abscess, furuncle and carbuncle of buttock | Skin | * | <0.04% | 0 | 0.00% |
| L02.4 | Cutaneous abscess, furuncle and carbuncle of limb | Skin | 5 | 0.04% | 0 | 0.00% |
| L02.8 | Cutaneous abscess, furuncle and carbuncle of other sites | Skin | 0 | 0.00% | * | <0.04% |
| L02.9 | Cutaneous abscess, furuncle and carbuncle, unspecified | Skin | * | <0.04% | * | <0.04% |
| L03.1 | Cellulitis of other parts of limb | Skin | 78 | 0.68% | 82 | 0.63% |
| L03.3 | Cellulitis of trunk | Skin | * | <0.04% | 0 | 0.00% |
| L03.9 | Cellulitis, unspecified | Skin | 188 | 1.64% | 138 | 1.06% |
| L08.8 | Other specified local infections of skin and subcutaneous tissue | Skin | * | <0.04% | 0 | 0.00% |
| L08.9 | Local infection of the skin and subcutaneous tissue, unspecified | Skin | 9 | 0.08% | 10 | 0.08% |
| L30.3 | Infective dermatitis | Skin | 0 | 0.00% | * | <0.04% |
| M00.9 | Pyogenic arthritis, unspecified, multiple sites | B&J | 53 | 0.46% | 34 | 0.26% |
| M46.2 | Osteomyelitis of vertebra, site unspecified | B&J | 11 | 0.10% | * | <0.04% |
| M46.4 | Discitis, unspecified, site unspecified | B&J | 22 | 0.19% | 16 | 0.12% |
| M86.1 | Other acute osteomyelitis | B&J | * | <0.04% | * | <0.04% |
| M86.6 | Other chronic osteomyelitis | B&J | 13 | 0.11% | 10 | 0.08% |
| M86.8 | Other osteomyelitis | B&J | 5 | 0.04% | * | <0.04% |
| M86.9 | Osteomyelitis, unspecified | B&J | 129 | 1.13% | 64 | 0.49% |
| N10 | Acute tubulo-interstitial nephritis | GUI | 27 | 0.24% | 13 | 0.10% |
| N11.1 | Chronic obstructive pyelonephritis | GUI | * | <0.04% | 0 | 0.00% |
| N12 | Tubulo-interstitial nephritis, not specified as acute or chronic | GUI | 38 | 0.33% | 24 | 0.19% |
| N13.6 | Pyonephrosis | GUI | * | <0.04% | 5 | 0.04% |
| N15.1 | Renal and perinephric abscess | GUI | 11 | 0.10% | 6 | 0.05% |
| N15.9 | Renal tubulo-interstitial disease, unspecified | GUI | * | <0.04% | 0 | 0.00% |
| N30.0 | Acute cystitis | GUI | * | <0.04% | * | <0.04% |
| N30.8 | Other cystitis | GUI | * | <0.04% | * | <0.04% |
| N30.9 | Cystitis, unspecified | GUI | * | <0.04% | * | <0.04% |
| N39.0 | Urinary tract infection, site not specified | GUI | 926 | 8.10% | 866 | 6.68% |
| N41.2 | Abscess of prostate | GUI | * | <0.04% | 0 | 0.00% |
| N45.9 | Orchitis, epididymitis and epididymo-orchitis without abscess | GUI | * | <0.04% | 0 | 0.00% |
| N48.1 | Balanitis | GUI | * | <0.04% | 0 | 0.00% |
| N70.0 | Acute salpingitis and oophoritis | GUI | * | <0.04% | 0 | 0.00% |
| N70.9 | Salpingitis and oophoritis, unspecified | GUI | * | <0.04% | * | <0.04% |
| N71.9 | Inflammatory disease of uterus, unspecified | GUI | * | <0.04% | * | <0.04% |
| N73.1 | Chronic parametritis and pelvic cellulitis | GUI | * | <0.04% | 0 | 0.00% |
| N73.9 | Female pelvic inflammatory disease, unspecified | GUI | * | <0.04% | * | <0.04% |

* - Non-zero frequency 5 or less. † B&J = Bone & Joint, CNS = Central Nervous System, GIT = Gastrointestinal Tract, GUI = Genitourinary, LRTI = Lower Respiratory Tract, TB = Tuberculosis, UTRI = Upper Respiratory Tract. Skin includes Cellulitis. In the analysis, CNS, Eye, Mycoses, TB, and URTI were classed as “Other” as numbers were too small. ‡ - Percentage of infections deaths for each group. Note that the above table only shows codes with an occurrence. A fuller list of the infection codes searched can be found here: <https://doi.org/10.24376/rd.sgul.21565557>

## Table S10 – Crude annual mortality rates during 2015-9 by different underlying infectious causes in T2D and non-diabetes

| Underlying Cause | All ages (41-90) | | | Ages 41-75 | | | Ages 76-90 | | |
| --- | --- | --- | --- | --- | --- | --- | --- | --- | --- |
|  | T2D | Non-D | Difference | T2D | Non-D | Difference | T2D | Non-D | Difference |
|  |  |  |  |  |  |  |  |  |  |
| Bone/ Joint | 0.10 | 0.03 | *+0.07* | 0.05 | 0.01 | *+0.04* | 0.23 | 0.08 | *+0.15* |
| Gastrointestinal | 0.22 | 0.09 | *+0.13* | 0.11 | 0.03 | *+0.08* | 0.56 | 0.28 | *+0.28* |
| Genitourinary | 0.43 | 0.20 | *+0.23* | 0.16 | 0.04 | *+0.12* | 1.23 | 0.68 | *+0.55* |
| LRT - Pneumonia | 1.99 | 1.10 | *+0.89* | 0.68 | 0.25 | *+0.43* | 5.94 | 3.55 | *+2.39* |
| LRT - Other | 1.47 | 1.07 | *+0.41* | 0.71 | 0.39 | *+0.32* | 3.78 | 3.03 | *+0.75* |
| Sepsis (underlying) | 0.24 | 0.10 | *+0.14* | 0.14 | 0.04 | *+0.10* | 0.57 | 0.30 | *+0.28* |
| Sepsis (any) | 2.18 | 1.02 | *+1.16* | 1.16 | 0.37 | *+0.80* | 5.23 | 2.90 | *+2.33* |
| Skin / Cellulitis | 0.14 | 0.06 | *+0.08* | 0.09 | 0.02 | *+0.08* | 0.27 | 0.17 | *+0.10* |
| Other | 0.18 | 0.11 | *+0.07* | 0.10 | 0.05 | *+0.05* | 0.42 | 0.29 | *+0.12* |

Note: Mortality rates are per 1000-person years for people with Type 2 diabetes (T2D) and age-sex-ethnicity matched non-diabetes (Non-D) group, and correspond to bars plotted in Figure 2 in main paper. Difference is T2D rate minus Non-D rate.

## Figure S1 – Summary of selected study participants with type 2 diabetes, and matched group without any diabetes

**568,645 (6.5%)**
initially classed with diabetes prior to 2015*

**8,722,348 (100%)**Patients aged 18-90 active on CPRD on 1^st^ January 2015 and registered for > 1 year

**509,403**with Type 2 diabetes aged 41-90 only

**527,151 (6.0%)**with Type 2 diabetes

**976,431**without diabetes matched on age, sex and ethnicity

**85,367 (16.8%)**with linked ONS death during 2015-9

**106,824 (10.9%)**with linked ONS death during 2015-9

CPRD = Clinical Practice Research Datalink. ONS = Office for National Statistics.

*Read code lists for diabetes are available in the repository <https://doi.org/10.24376/rd.sgul.21565557.v1>

## Figure S2 – Age-specific hazard ratios and risk ratios for all-cause mortality during 2015-9 in T2D vs non-diabetes, all patients


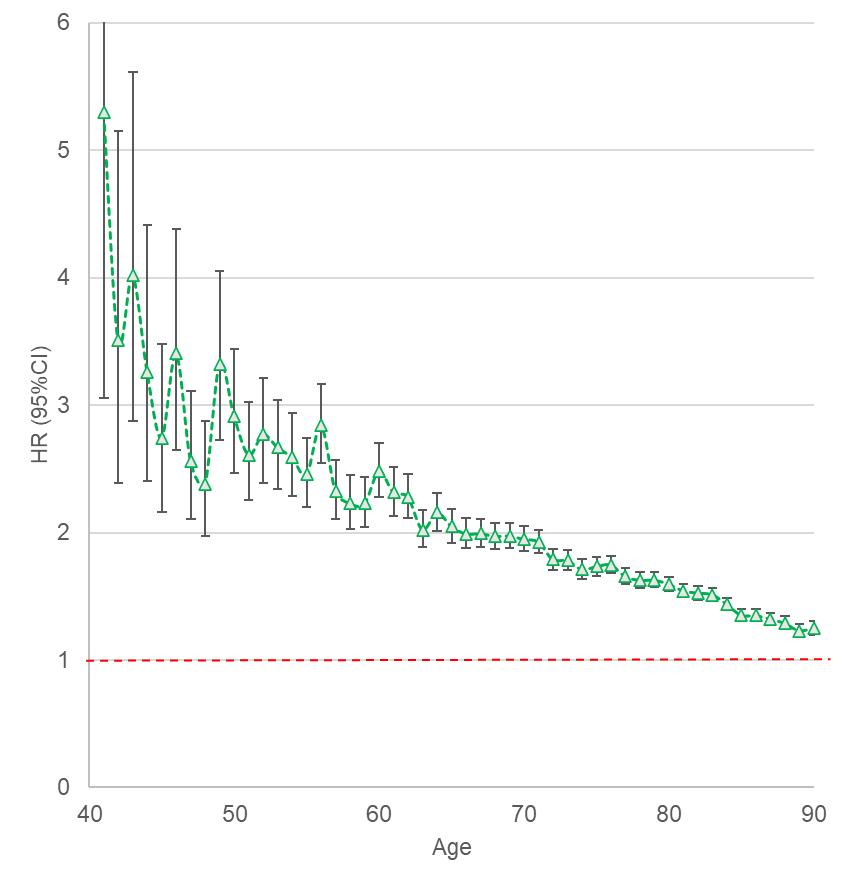

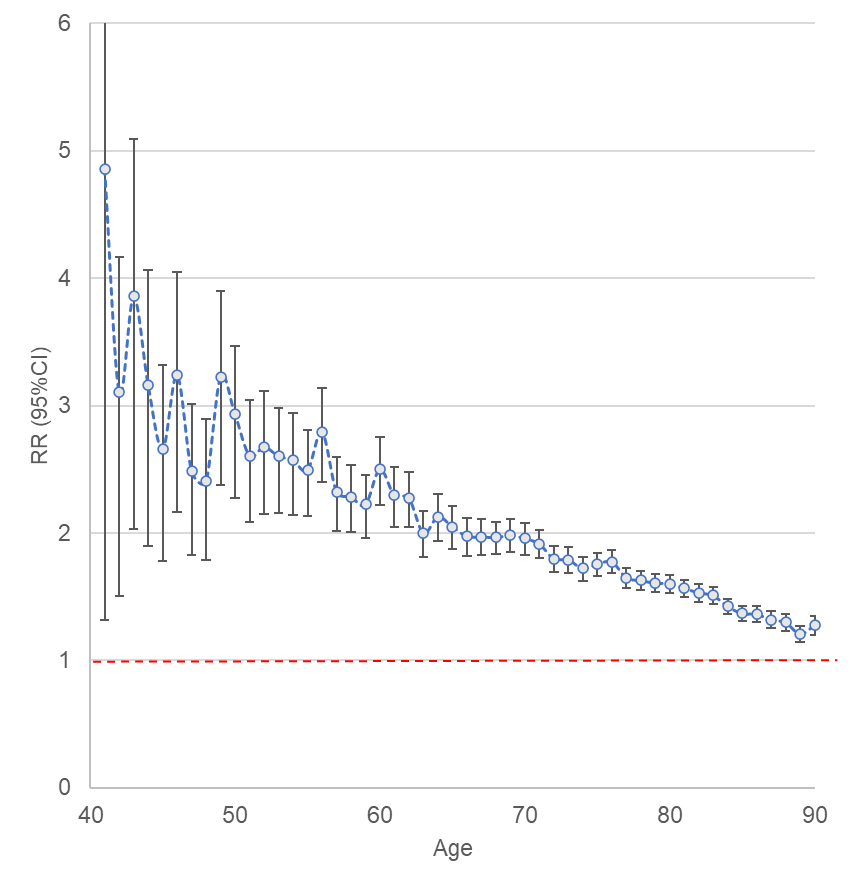


Green Triangles = Hazard ratio (and 95% confidence intervals) from Cox model, stratified by match set, fitted to each age separately and adjusted for region. Blue Circles = Relative risk (and 95% confidence intervals) from GEE Poisson model, accounting for clustering by match set, adjusted for sex, ethnicity and region.

## Figure S3 – Cause of death during 2015-9 in T2D and non-diabetes, by different categorisations


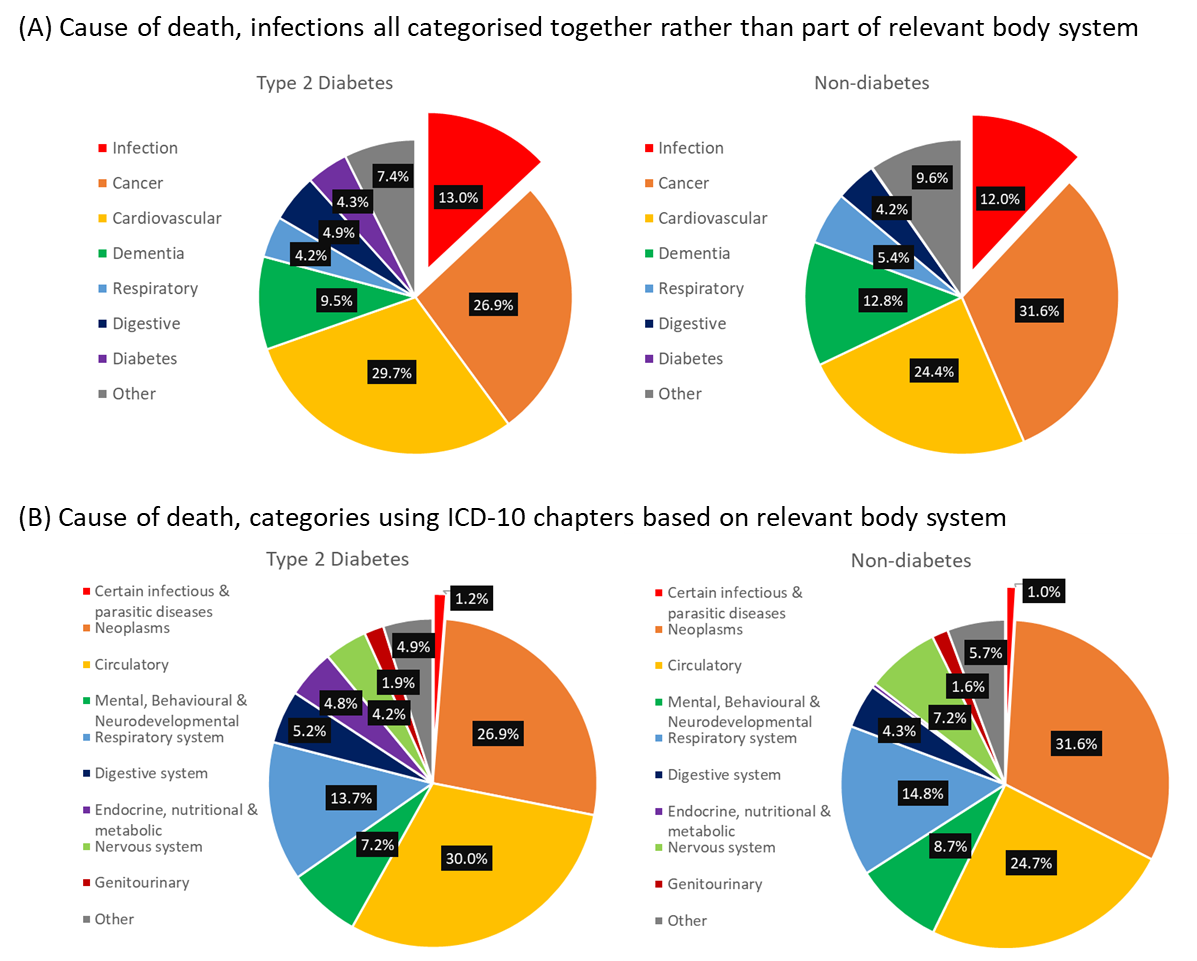


## Figure S4 – Crude mortality rates and hazard ratios by cause of death during 2015-9 in T2D vs non-diabetes by ethnicity


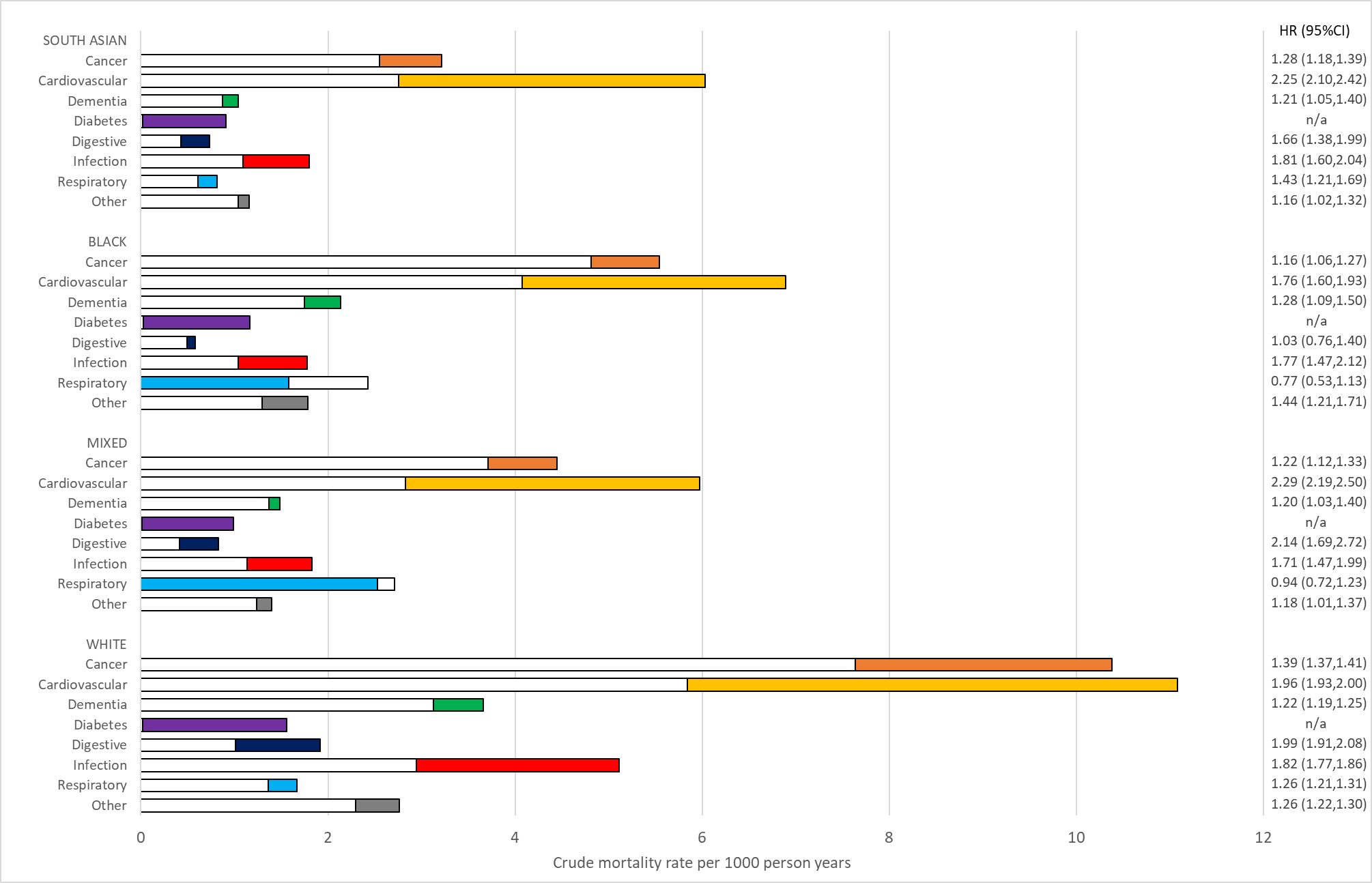


Note: White bars = crude mortality rate in non-diabetes. Coloured bars = excess crude mortality rate in people with T2D. Combined bar (white + colour) = crude mortality rate in T2D. The exception is for respiratory among Black & Mixed group, where mortality rate is lower in people with T2D and the reverse colour scheme is used.

## Figure S5 – Percentage of deaths during 2015-9 with infection recorded as underlying cause, any mention of sepsis or infection as contributory cause only in T2D vs non-diabetes, ages 41-90.


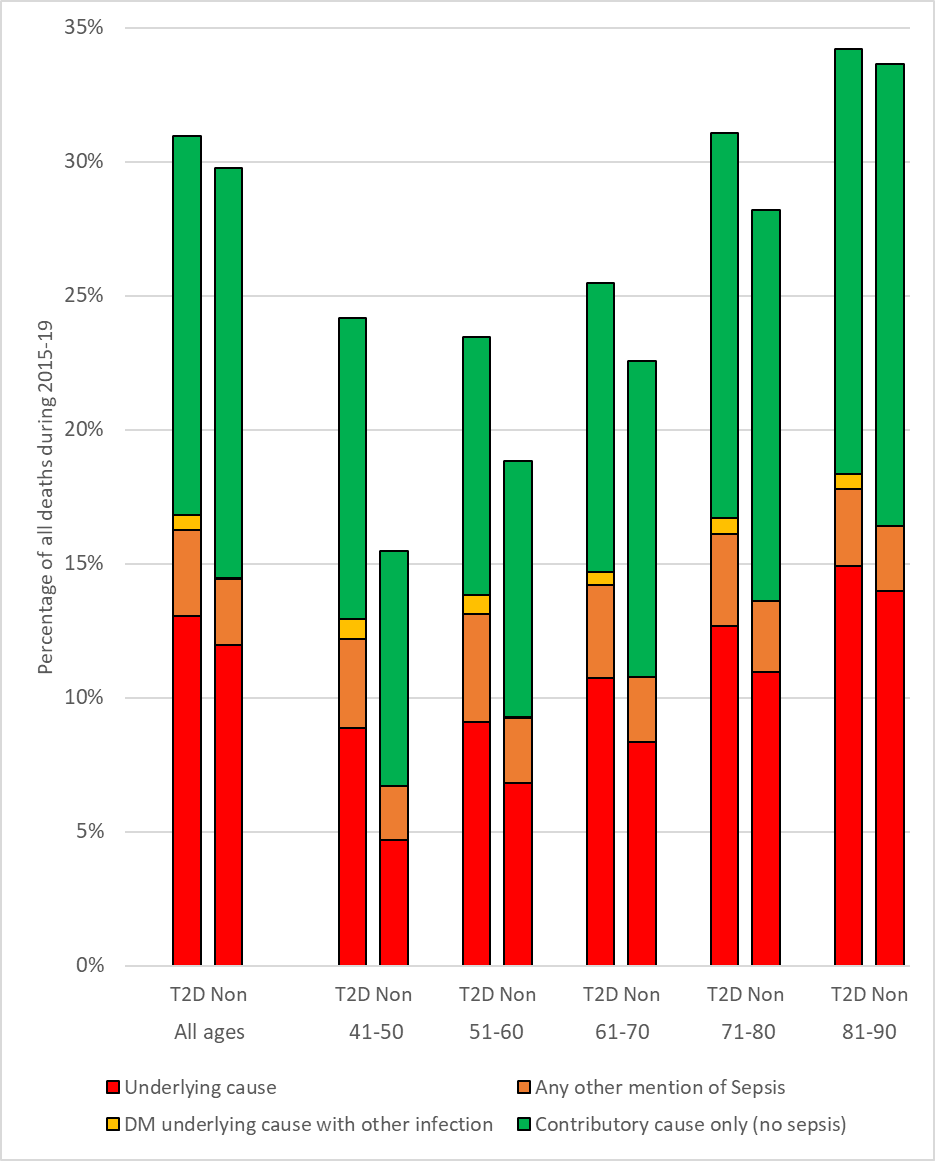

Supplement: Supplementary Material Revised [file mmc1.docx]
